# A multi-scale analysis of bull sperm methylome revealed both species peculiarities and conserved tissue-specific features

**DOI:** 10.1186/s12864-018-4764-0

**Published:** 2018-05-29

**Authors:** Jean-Philippe Perrier, Eli Sellem, Audrey Prézelin, Maxime Gasselin, Luc Jouneau, François Piumi, Hala Al Adhami, Michaël Weber, Sébastien Fritz, Didier Boichard, Chrystelle Le Danvic, Laurent Schibler, Hélène Jammes, Hélène Kiefer

**Affiliations:** 1 UMR BDR, INRA, ENVA, Université Paris Saclay, 78350 Jouy en Josas, France; 2 Present Address: Laboratory of Animal Reproduction, Department of Biological Sciences, Faculty of Science and Engineering, University of Limerick, Limerick, Ireland; 3 ALLICE, 149 rue de Bercy, 75012 Paris, France; 4 Present Address: Institut Curie, PSL Research University, CNRS, UMR3664, 75005 Paris, France; 5 Present Address: Sorbonne Universités, UPMC Univ Paris 06, CNRS, UMR3664, 75005 Paris, France; 6 CNRS, Université de Strasbourg, UMR7242 Biotechnologie et signalisation cellulaire, 300 bd Sébastien Brant, 67412 Illkirch cedex, France; 7 UMR GABI, INRA, AgroParisTech, Université Paris Saclay, 78350 Jouy en Josas, France; 8 UMR CNRS/USTL 8576, UGSF, Villeneuve D’Ascq, France

**Keywords:** DNA methylation, Sperm, Cattle, Satellite repeats

## Abstract

**Background:**

Spermatozoa have a remarkable epigenome in line with their degree of specialization, their unique nature and different requirements for successful fertilization. Accordingly, perturbations in the establishment of DNA methylation patterns during male germ cell differentiation have been associated with infertility in several species. While bull semen is widely used in artificial insemination, the literature describing DNA methylation in bull spermatozoa is still scarce. The purpose of this study was therefore to characterize the bull sperm methylome relative to both bovine somatic cells and the sperm of other mammals through a multiscale analysis.

**Results:**

The quantification of DNA methylation at CCGG sites using luminometric methylation assay (LUMA) highlighted the undermethylation of bull sperm compared to the sperm of rams, stallions, mice, goats and men. Total blood cells displayed a similarly high level of methylation in bulls and rams, suggesting that undermethylation of the bovine genome was specific to sperm. Annotation of CCGG sites in different species revealed no striking bias in the distribution of genome features targeted by LUMA that could explain undermethylation of bull sperm. To map DNA methylation at a genome-wide scale, bull sperm was compared with bovine liver, fibroblasts and monocytes using reduced representation bisulfite sequencing (RRBS) and immunoprecipitation of methylated DNA followed by microarray hybridization (MeDIP-chip). These two methods exhibited differences in terms of genome coverage, and consistently, two independent sets of sequences differentially methylated in sperm and somatic cells were identified for RRBS and MeDIP-chip. Remarkably, in the two sets most of the differentially methylated sequences were hypomethylated in sperm. In agreement with previous studies in other species, the sequences that were specifically hypomethylated in bull sperm targeted processes relevant to the germline differentiation program (piRNA metabolism, meiosis, spermatogenesis) and sperm functions (cell adhesion, fertilization), as well as satellites and rDNA repeats.

**Conclusions:**

These results highlight the undermethylation of bull spermatozoa when compared with both bovine somatic cells and the sperm of other mammals, and raise questions regarding the dynamics of DNA methylation in bovine male germline. Whether sperm undermethylation has potential interactions with structural variation in the cattle genome may deserve further attention.

**Electronic supplementary material:**

The online version of this article (10.1186/s12864-018-4764-0) contains supplementary material, which is available to authorized users.

## Background

Sperm unique morphology and functions result from a long differentiation process that requires dynamic epigenetic reprogramming of the genome [1], which starts with the global erasure and reestablishment of DNA methylation marks in fetal and post-natal germ cells [2] and continues throughout adulthood. The maintenance of DNA methylation, the accumulation of non-coding RNAs, the implementation of post-translational histone modifications or sperm-specific variants and finally histone-to-protamine replacement then occur progressively during the sequential mitosis, meiosis, differentiation and maturation steps of spermatogenesis [3, 4]. The reorganization of epigenetic marks during spermatogenesis enables a dramatic compaction of the sperm nucleus, thus improving motility and DNA damage protection in the female genital tract, and plays a fundamental role in subsequent development of the embryo [5]. Alterations to the epigenetic reprogramming of the male germline may potentially affect sperm functions and fertilization efficiency [6], and numerous studies have reported associations between an abnormal sperm epigenome and a low sperm count or sperm dysmorphia, fertilization failures, poor embryogenesis, low pregnancy outcomes and metabolic disorders affecting the offspring [7–17]. Accordingly, studies in human cohorts [12, 18, 19] and genetic or pharmacological alterations to DNA methylation in mice [20–22] have emphasized the prominent role of DNA methylation in male germ cell differentiation and male fertility.

Comparatively, studies on DNA methylation in bovine spermatozoa are still scarce, and have often focused on candidate loci [23–25]. Recent genome-wide studies have identified sperm DNA methylation marks associated with subfertility in buffalo and bulls [26, 27], as well as regions that are hypermethylated in sperm relative to the embryo, studied using a platform dedicated to small samples [28]. However, a comprehensive view of the sperm methylome in bovine species is still lacking, even though this knowledge could enable promising advances in the cattle industry. Indeed, domestication, the creation of highly specialized breeds and decades of genetic improvement have shaped the bovine genome [29]. This undoubtedly has also had a profound impact on the methylome, since DNA methylation is directly affected by the CpG content of the genome and its alteration by DNA polymorphism [30]. Whether these changes are of functional significance and contribute to the establishment of phenotypes needs to be ascertained. In addition, in a context of genomic selection, more information on the epigenetic features transferred to the embryo alongside the paternal genetic heritage is necessary in order to improve semen quality control procedures as well as to guarantee semen fertility and proper embryo development.

In order to contribute knowledge in this field, we established a thorough description of the methylome of bull spermatozoa at different scales, using luminometric methylation assay (LUMA), methylated DNA immunoprecipitation (MeDIP), reduced representation bisulfite sequencing (RRBS) and pyrosequencing. We report here on the global DNA methylation level of bull sperm relative to both bovine somatic cells and the sperm of other mammals, and on a comparison of genome-wide methylation patterns between bovine sperm and somatic cells.

## Methods

### Animals and cell/tissue collection

All study methods were implemented in accordance with EU guidelines and regulations (Directive 2010/63/UE). For animals maintained in INRA facilities, the experimental protocols were approved by the INRA local Ethics Committee (COMETHEA, authorization numbers 12/160 and Méjusseaumes Animal Care committee 0162503). The bull samples originated from bulls selected for artificial insemination and were provided by commercial companies: Montbéliarde breed by GEN’IATEST (France) and UMOTEST (France), Holstein and Normande breeds by EVOLUTION (France) and Belgian White Blue breed by AWE (Belgium). Other bovine tissues were collected from Holstein cows maintained at the INRA experimental farm (UCEA, INRA, France). The ram, goat and boar semen and blood samples were supplied by commercial companies (OSON, Capgenes and LNCR, respectively, France). Mice semen samples were collected from the caudal epididymis of 7-week-old male C57Bl/6JOlaHsd mice supplied by Harlan Laboratory (Netherlands) and euthanized by cervical dislocation. Stallion semen was supplied by Dr. M. Magistrini (UMR INRA 0085 PRC, France). Human sperm samples originated from patients included in a PHRC METASPERME study, coordinated by Dr. R. Levy (Laboratoire d’Histologie Embryologie Cytogénétique CECOS, Hôpital Jean Verdier, France); this study received the approval from French ethics board (Conseil d’évaluation éthique pour les recherches en santé, CERES) and all the patients gave their informed written consent to participate.

**Fig. 1:**
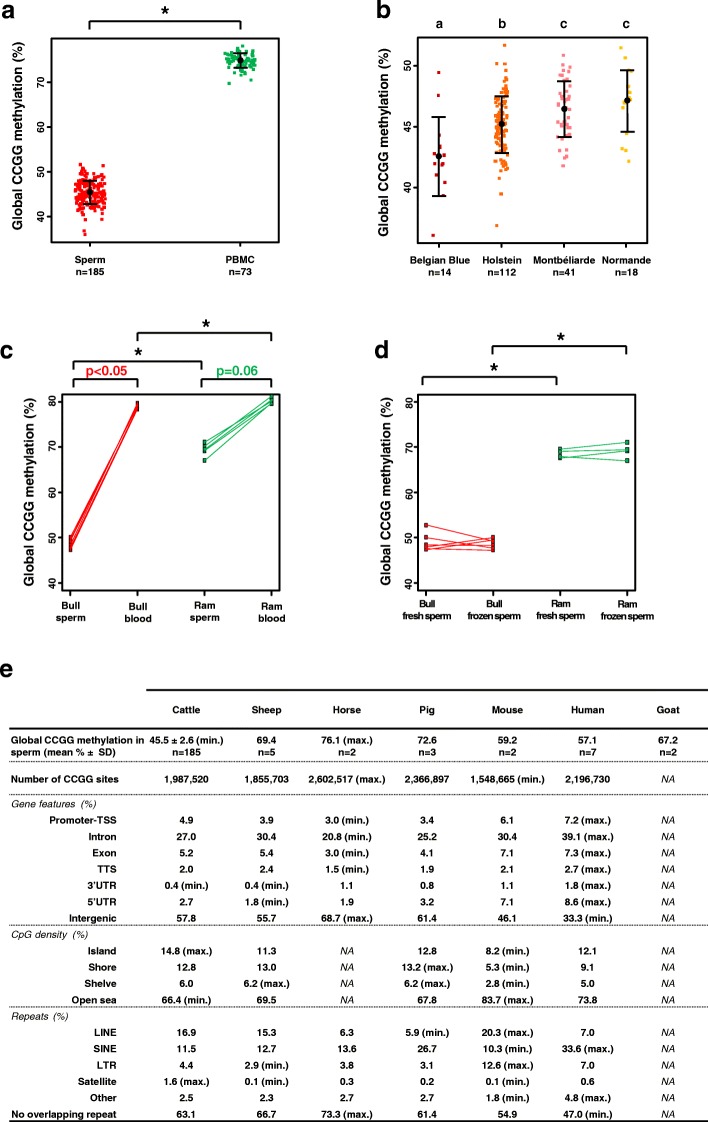
Global DNA methylation level measured by LUMA is low in bovine sperm. **a** Global DNA methylation level in bovine sperm and PBMCs. Each colored dot represents one individual. The black dots and horizontal bars indicate the means ± standard deviations. The difference between cell types is highly significant (*p* < 2.2e-16, Welch’s t-test). **b** Global DNA methylation level of sperm in four bovine breeds. The effect of the breed on CCGG methylation is significant (*p* < 0.05, one-way analysis of variance for independent samples). Significant differences between breeds are indicated by different letters (*p* < 0.05, multiple comparisons of means using Tukey’s test). **c**, **d** Global DNA methylation level in bull and ram samples. Significant differences between independent samples are indicated by asterisks (*p* < 0.05, permutation test), while paired samples are connected by plain lines. **c** Global DNA methylation level in blood cells and semen for bulls (*n* = 6) and rams (*n* = 5). The *p*-values are indicated in red (bulls) and green (rams) for comparisons involving samples collected from the same individuals (permutation test for paired samples). **d** Global DNA methylation level in bull (*n* = 6) and ram (*n* = 4) sperm cells from fresh and frozen semen. The difference between fresh and frozen semen is not significant. **e** Global sperm DNA methylation level and CCGG distribution in several mammalian species. The CCGG sites were annotated relative to gene features, CpG density and overlapping repeats. For each genomic feature examined, species with extreme values are indicated (min. and max.). In the bovine genome, CCGG sites are particularly enriched in CpG islands and satellites, and are within the ranges of other species for other genomic features. SD: standard deviation

In Fig. 1c-d, semen and blood from the same individuals were collected using standard procedures on bulls and rams maintained in semen production centers. Total blood was used for DNA extraction. For both bulls and rams, collected semen was extended with Optidyl (Cryo-Vet) and either underwent direct DNA extraction (fresh semen) or was subjected to standard techniques for semen processing (straw conditioning, freezing and storage in liquid nitrogen; frozen semen). Other bull semen samples were in the form of frozen straws stored in liquid nitrogen.

For all semen samples except those from humans and stallions, the possible contamination of spermatozoa by somatic cells was checked systematically under the microscope and confirmed to be below detectable levels. The human and stallion semen samples contained observable somatic cells and were therefore processed as previously described to ensure the absence of potential contamination: the human semen samples were subjected to a stringent somatic cell lysis protocol [18] and the sperm from stallions were purified by single layer centrifugation using Androcoll-E-Large (SLU, Uppsala, Sweden) [31].

Primary cultures of fibroblasts were derived from ear skin biopsies from three separate adult heifers and cultured until passage 11 in Dulbecco’s modified Eagle medium supplemented with 10% fetal calf serum and 1% penicillin-streptavidin (Life Technologies) at 38 °C with 5% CO_2_. Livers were obtained from adult cows slaughtered at the INRA experimental facilities. Peripheral blood mononuclear cells (PBMCs) were isolated from blood collected from the jugular vein and centrifuged using a Ficoll gradient. To obtain the monocyte fraction, PBMCs were incubated in the presence of microbeads conjugated to monoclonal anti-CD14 antibodies (mouse IgG2a; Miltenyi Biotec) in MACS BSA buffer, for 15 min. at 4 °C under gentle agitation. Magnetic separation was then performed using MS Columns following the manufacturer’s instructions. The tissues and cells were snap-frozen in liquid nitrogen and stored at − 80 °C until DNA extraction.

The sample types and experiments performed are summarized in Table 1.

**Table 1:** Samples and experiments

Experiment	Species	Breed/strain	Sample type	Sample number	Figures
LUMA	Cattle	Holstein	PBMCs	73	Fig. 1a
	Cattle	Belgian White Blue	Sperm, frozen	14	Fig. 1a-b, e
	Cattle	Holstein	Sperm, frozen	112	Fig. 1a-b, e
	Cattle	Montbéliarde	Sperm, frozen	41	Fig. 1a-b, e
	Cattle	Normande	Sperm, frozen	18	Fig. 1a-b, e
	Cattle	Montbéliarde	Sperm, frozen	6	Fig. 1c-d
	Cattle	Montbéliarde	Total blood	6	Fig. 1c
	Sheep	Ile-de-France	Sperm, frozen	5	Fig. 1c-d, e
	Sheep	Ile-de-France	Total blood	5	Fig. 1c
	Cattle	Montbéliarde	Sperm, fresh	6	Fig. 1d
	Sheep	Ile-de-France	Sperm, fresh	4	Fig. 1d
	Horse	Welsh	Sperm, fresh	2	Fig. 1e
	Pig	mixed	Sperm, fresh	3	Fig. 1e
	Mouse	C57Bl/6JOlaHsd	Sperm, fresh	2	Fig. 1e
	Human		Sperm, frozen	7	Fig. 1e
	Goat	Alpine	Sperm, frozen	2	Fig. 1e
Genotyping	Cattle	Montbéliarde	Sperm, fresh	2	Additional file 1: Figure S2
	Cattle	Montbéliarde	Total blood	2	Additional file 1: Figure S2
MeDIP	Cattle	Holstein	Sperm, frozen	4	Figs. 2, 3, 4, 6
	Cattle	Holstein	Liver	4	Figs. 2, 3, 4, 6
	Cattle	Holstein	Fibroblasts	3	Figs. 2, 3, 4, 6
RRBS	Cattle	Holstein	Sperm, frozen	2	Figs. 2, 3, 5, 6
	Cattle	Holstein	Monocytes	2	Figs. 2, 3, 5, 6
	Cattle	Holstein	Fibroblasts	2	Figs. 2, 3, 5, 6

All the samples were independent, except for (i) the sperm and blood samples in Fig. 1c-d that were collected on the same bulls and rams, and (ii) two fibroblast samples and two livers that were collected on the same animals. Two independent amplifications of the same fibroblast cultures were used for MeDIP and RRBS. The sperm and blood DNA samples used for genotyping were the same as used for LUMA, Fig. 1c-d. PBMCs: peripheral blood mononuclear cells

### Genomic DNA extraction and genotyping

One straw of bull semen was used for DNA extraction (about 20 million spermatozoa). After thawing at 37 °C, the semen was washed with phosphate buffer saline (PBS) to remove the extender, and incubated overnight at 55 °C in 200 μl lysis buffer (10 mM Tris-HCl pH 7.5, 25 mM EDTA, 1% SDS, 75 mM NaCl, 50 mM dithiothreitol (DTT) and 0.5 μg glycogen) in the presence of 0.2 mg/ml proteinase K. After incubation with 25 μg/ml RNAse A for 1 h at 37 °C, genomic DNA was extracted twice using phenol and phenol:chloroform (1:1), then ethanol precipitated and washed. The dried pellet was re-suspended in TE buffer (10 mM Tris HCl pH 7.5, 2 mM EDTA) and the DNA concentration was measured using a Qubit 2.0 Fluorometer (Invitrogen). Fresh bull semen, total blood from bulls and rams, fresh or frozen semen from rams, boars, mice, goats, processed semen from stallions and men were treated in an identical manner as the frozen straws from bull semen.

DNA extraction from liver samples was performed as described elsewhere [32]. The same procedure was used for fibroblasts and monocytes, except that the cells were lysed by the direct addition of lysis buffer and proteinase K to the cell pellet.

The genotyping of two Montbéliarde bulls was performed by LABOGENA (France) on semen and blood DNA from the same individuals using the commercially available BovineSNP50 v2 BeadChip (Illumina). Genotypes were determined using the Genotyping Module of GenomeStudio software (Illumina). For each animal, copy number variations (CNVs) were searched for and compared between tissues. The Log R Ratio (LRR, normalized measurement of total signal intensity) and B Allele Frequency (BAF, measurement of the allelic intensity ratio) were used to infer copy number changes in the genome. For example, in the presence of a deletion, LRR values increase and BAF values cluster around 0 or 1 but are absent at around 0.5, due to a lack of heterozygotes. LRR and BAF were then plotted along the genome and compared between tissues.

### In silico analyses

A script developed in house was used to extract the coordinates of all the CCGG sites present in the genomes of different species (cattle, sheep, horse, pig, mouse and human; Fig. 1e). The CCGG sites were then annotated relative to different gene features, CpG density and repeats by means of a pipeline developed in house (https://github.com/FAANG/faang-methylation/tree/master/RRBS-toolkit/Annotation) and using the genome annotation features indicated in Additional file 1: Table S1. The following criteria were applied: promoter-TSS, − 1000 to + 100 bp relative to the transcription start site (TSS); TTS: -100 to + 1000 bp relative to the transcription termination site (TTS); shore, up to 2000 bp from a CpG island (CGI); and shelve up to 2000 bp from a shore. A site/fragment was considered to belong to a CGI (respective shore and shelve) if an overlap of at least 75% was observed between the site/fragment and the CGI (respective shore and shelve). A site/fragment was considered as being overlapped by a repetitive element whatever the extent of this overlapping.

In Additional file 1: Tables S2 and S3, in silico reduced representation (RR) genomes digested by MspI restriction enzyme were produced for different species and using different size selection criteria by means of another pipeline developed in house (https://github.com/FAANG/faang-methylation/tree/master/RRBS-toolkit/RR_genome). The RR genome fragments were then annotated as explained above.

### Luminometric methylation assay (LUMA)

Global DNA methylation levels were quantified using LUMA, as previously described [33, 34]. Briefly, 1 μg of genomic DNA was cleaved using the isochizomeres HpaII (methylation sensitive) and MspI (non-methylation-sensitive) in two separate reactions and in the presence of EcoRI to standardize for DNA amounts. The three enzymes were purchased from New England Biolabs. The protruding ends were then used as templates for pyrosequencing with the Pyromark Q24 device and Pyromark Gold Q96 reagents (Qiagen). The luminometric signals produced by either the sequential incorporation of C and G nucleotides (reflecting the number of CCGG sites digested by HpaII or MspI) or the sequential incorporation of A and T nucleotides (reflecting the number of AATT sites digested by EcoRI), were then quantified using Pyromark Q24 software. Each sample was assayed in duplicate. The global methylation percentage per sample was then calculated as follows:

$$ \mathrm{Methylation}\%=100-\frac{\mathrm{Average}\ \mathrm{signal}\ \mathrm{obtained}\ \mathrm{with}\ \mathrm{HpaII}\ \mathrm{after}\ \mathrm{EcoRI}\ \mathrm{normalization}}{\mathrm{Average}\ \mathrm{signal}\ \mathrm{obtained}\ \mathrm{with}\ \mathrm{MspI}\ \mathrm{after}\ \mathrm{EcoRI}\ \mathrm{normalization}}\ast 100 $$

The conditions were compared using non-parametric tests suited to small samples (permutation tests for two independent samples or for two paired samples according to the situation, with Monte-Carlo sampling of 100,000 permutations) or using t-test and analysis of variance when appropriated (larger samples with a normal distribution).

### Methylated DNA immunoprecipitation (MeDIP), microarray hybridization and data analysis

MeDIP and quality controls by PCR were performed as described elsewhere [32]. The antibody used for immunoprecipitation was BI-MECY-1000 5-methylcytidine antibody (Eurogentec). To prevent any technical bias, the products of five independent MeDIP experiments were pooled for each sample. After moderate genome amplification, the pooled MeDIP reactions and corresponding input DNA were labelled with Cy3 and Cy5 and hybridized on a Roche-NimbleGen 3x720K microarray, with technical dye-swaps for every sample. The microarray targeted the promoter region (− 2000 to + 1360 bp relative to the gene start) of 21,296 bovine genes, according to an annotation file downloaded from the Johns Hopkins University Center for Computational Biology FTP website (ftp://ftp.ccb.jhu.edu/pub/data/assembly/Bos_taurus/Bos_taurus_UMD_3.0/annotation/; accessed Aug. 2010). The microarray design and hybridization protocol, as well as more details on the data analysis, can be found in [35]. The identification of regions of interest containing clusters of probes enriched in at least one tissue, the identification of differentially methylated regions (DMRs) among these regions of interest, as well as the calculation of mean percentages of enriched probes (Pr) in each tissue for each region r (DMR or region of interest), are detailed in the Additional file 1: Supplementary methods. Three Pr were obtained per region: Pr_sperm_, Pr_liver_ and Pr_fibroblasts_. The scatterplot shown in Fig. 3d illustrates the Pr_liver_ - Pr_sperm_ and Pr_fibroblasts_ - Pr_sperm_ differences for the regions of interest (in black) and for the DMRs specific to the comparison between sperm and somatic cells (in red). Positive values for both Pr_liver_ - Pr_sperm_ and Pr_fibroblasts_ - Pr_sperm_ indicated that the two somatic cell types were more methylated than sperm in the region considered, while negative values for both Pr_liver_ - Pr_sperm_ and Pr_fibroblasts_ - Pr_sperm_ indicated the contrary. Similarly, an opposite sign for the values of Pr_liver_ - Pr_sperm_ and Pr_fibroblasts_ - Pr_sperm_ reflected the contrasting behaviors of liver and fibroblasts. Among the DMRs, those undermethylated in sperm were selected based on a positive value for both Pr_liver_ - Pr_sperm_ and Pr_fibroblasts_ - Pr_sperm_ and were annotated as explained regarding the in silico analyses, together with the 27,684 regions of interest. Genes containing DMRs were subjected to DAVID analysis (*Database f*or Annotation, Visualization and Integrated Discovery; [36]) using genes containing the 27,684 regions of interest as the background.

### Reduced representation bisulfite sequencing (RRBS) and data analysis

RRBS libraries were prepared as described elsewhere [37, 38]. Briefly, 200 ng of genomic DNA were digested by MspI (Thermo Scientific), end-repaired and ligated to 55 bp Illumina adapters for paired-end sequencing. Size selection by gel excision was performed in order to select fragments ranging from 150 to 400 bp (genomic fragments of 40-290 bp + adapters). The DNA was then purified using the MinElute gel extraction kit (Qiagen) and then bisulfite-converted twice consecutively with the EpiTect bisulfite kit (Qiagen), following the manufacturer’s instructions for DNA extracted from FFPE tissues. Converted DNA was amplified with Pfu Turbo Cx hotstart DNA polymerase (Agilent) using 14 PCR cycles for sperm and fibroblasts and 12 cycles for monocytes. The libraries were then purified using Agencourt Ampure beads (Beckman-Coulter) and sequenced on an Illumina HiSeq2500 sequencer to produce 75 bp paired-end reads (Integragen SA, France).

RRBS sequences were analyzed using an integrated pipeline combining scripts developed in house in Python, R and Shell, together with external tools (https://github.com/FAANG/faang-methylation/tree/master/RRBS-toolkit/). Details about the analysis and the identification of differentially methylated cytosines (DMCs) are provided in the Additional file 1: Supplementary methods.

For each tissue, the mean methylation percentage was calculated (mean of the methylation percentages obtained in the two biological replicates) as well as the difference between two tissues (Additional file 2, column L). The scatterplot shown in Fig. 3e illustrates the differences between monocytes and sperm and between fibroblasts and sperm for the 1,580,644 CpGs covered by 5 to 500 uniquely mapped reads (CpGs 5-500) in all six samples (in black) and for the DMCs specific to the comparison between sperm and somatic cells (in red). Among these DMCs, those undermethylated in sperm were selected based on a positive value for both differences. Together with the CpGs 5-500, they were then annotated relative to gene features, CGIs and repeats as explained for the in silico analyses. Genes containing DMCs were subjected to DAVID analysis using genes containing the 1,580,644 CpGs 5-500 as the background.

To better characterize repetitive elements, an artificial genome containing the consensus sequence of each bovine repeat was constituted from the Repbase database [39]. Reads were aligned on this artificial genome as explained above, and the average methylation percentage was calculated for each repeat and each sample (average methylation percentage for all CpGs included in one genomic repeat and covered by either 5-500 reads or by > 500 reads).

### Bisulfite-pyrosequencing

Bisulfite conversion was performed on 1 μg genomic DNA as described elsewhere [40]. After ethanol precipitation, the DNA pellet was re-suspended in 20 μl H_2_O.

For *LSM4* and *BTSAT4*, primers were designed using the MethPrimer program [41] and amplifications were carried out from 1 μl treated DNA with Platinum Taq DNA polymerase (Invitrogen), according to the manufacturer’s instructions with variable MgCl_2_ concentrations. The following program was used: 3 min. at 94 °C followed by 50 cycles of 30 s. at 94 °C, 1 min. at variable hybridization temperatures, 1 min. at 72 °C, and finally 10 min. at 72 °C. For *DDX4* and *SYCP3*, primers were designed using the Pyromark assay design software (Qiagen) and amplifications were performed using the Pyromark PCR kit (Qiagen) according to the manufacturer’s instructions. The primers used to amplify each region are listed in Additional file 1: Table S4, together with the hybridization temperatures and MgCl_2_ concentrations. The reverse primers were 5′-biotinylated.

After denaturation and purification, the biotinylated antisense strand of PCR product was used as a template for pyrosequencing with 0.3 μM pyrosequencing primer, using the Pyromark Q24 device and Pyromark Gold Q96 reagents (Qiagen). The pyrosequencing primers are listed in Additional file 1: Table S5. Each CpG was assayed in duplicate, and inconsistent duplicates (more than 5% difference) were repeated. The methylation percentage per CpG was then obtained by calculating the mean of all replicates that passed quality control by the Pyromark Q24 software. The statistical analysis was performed on the mean percentage per CpG using permutation tests as explained for LUMA.

## Results

### Global DNA methylation level is low in bull sperm

We first assessed the global level of DNA methylation in bull sperm relative to somatic cells (PBMCs) using LUMA [33, 34] on a large sample size. The average methylation at CCGG sites was dramatically lower in sperm (45.5%, *n* = 185) than in PBMCs (74.8%, *n* = 73; Fig. 1a). The standard deviations in this large sample were weak (2.6% for sperm and 1.6% for PBMCs), demonstrating the limited inter-individual variability within each cell type and the reliability of the technique to assess global DNA methylation. Because the sperm samples were collected from Holstein, Montbéliarde, Normande and Belgian White Blue bulls, we investigated the effect of the breed on global sperm DNA methylation. Methylation was significantly lower in Belgian White Blue than in any other breed and significantly lower in Holstein than in Normande and Montbéliarde (Fig. 1b). A bootstrap analysis confirmed that these breed-related differences were not due to an unbalanced number of bulls from each breed (Additional file 1: Figure S1). These results demonstrated that global methylation varied across different bovine breeds, suggesting that the presence of DNA polymorphism could influence the global CCGG content and methylation. However, the range of variation (from 42.6% in Belgian White Blue to 47.1% in Normande) was weak relative to the 30% difference we observed between sperm and PBMCs, suggesting that sperm weaker methylation was not breed-dependent. To determine whether the global DNA methylation of sperm was comparably low in another species, we collected paired semen and blood samples from bulls and rams. While sperm was less methylated than blood in both species (Fig. 1c), the difference between the two cell types was much greater for bulls (30% less methylation in sperm than in blood) than for rams (only 10% less methylation in sperm than in blood). Differences between species were observed in both cell types, but were broader for sperm (20% difference) than for blood (only 1.2% difference). In sperm, the difference between species was independent of the cryopreservation process (Fig. 1d).

This lower methylation of sperm may have resulted from a biased representation of the regions present in our sperm genomic DNA. Indeed, the high level of sperm chromatin compaction impeded the complete extraction of genomic DNA using standard procedures, which were therefore optimized by the addition of reducing agents such as DTT (see for instance [42]). The protocol we used during this study performed well for DNA extraction from human sperm (50 mM DTT, [7]). To investigate whether these conditions were also suited to bovine sperm, we used genomic DNA from two paired sperm and blood samples (the same samples as in Fig. 1c), both of which were purified in the presence of 50 mM DTT, as a template for genotyping. As expected, the same genotype was obtained from blood and sperm DNA in each bull. Likewise, for each animal, CNV profiles were compared between tissues because any difference might be indicative of preferential extraction. The plots were similar and no gross discrepancies could be observed between tissues (Additional file 1: Figure S2). This result therefore ruled out the possibility that some specific regions of the bovine genome failed to be extracted from sperm chromatin under our experimental conditions.

Because the genomic distribution of CCGG sites may display species-specific variations that might influence the methylation results, we compared sperm CCGG methylation in several species relative to the genomic features available. In the horse, pig, mouse, human and goat, sperm DNA methylation level was much higher than that measured in bovine and closer to that determined in sheep (Fig. 1e). The CCGG distribution in the bovine genome was within the range observed in other species for most of the features examined, except for CGIs that accounted for 14.2% of total CCGG sites in bovine vs. 8.0 to 12.2% in other species. The important representation of bovine CCGGs in CGIs was balanced by a low representation in open sea (regions with a low CpG density). Most mammalian CGIs are unmethylated in somatic cells and can become methylated during development and disease [43]. Because blood cells (in which most CGIs are supposed to be unmethylated) displayed a roughly similar methylation level in bovine and sheep, it is unlikely that the lower level of DNA methylation in bull sperm resulted solely from the higher percentage of bovine CCGG sites in CGIs. Another remarkable feature of the bovine CCGG sites was their strong enrichment in satellites, which represented 1.6% of all CCGGs vs. 0.1 to 0.6% in other species. However, whatever the species examined, the < 2% difference in CCGG sites present in satellites could not account for the > 10% difference in sperm methylation (Discussion).

Taken together, these data demonstrated that compared to somatic cells, bull sperm displayed a dramatically lower level of CCGG methylation which seemed to be specific to the bovine species. This lower methylation was neither related to the process of semen cryopreservation nor to a technical artefact, and could not be fully explained by the genomic distribution of CCGG sites in the bovine species.

### The tissue/cell type is a major determinant of DNA methylation landscapes in cattle

Because high-throughput analyses were necessary to identify regions that were hypomethylated in bovine sperm, we decided to assess two cost-effective approaches which are widely used to study DNA methylation: MeDIP-chip [44] and RRBS [37]. MeDIP-chip enables precise targeting of specific regions in the genome through custom design of the microarray (in our case, 3360 bp spanning the promoter and upstream region of each of the 21,296 bovine genes [35]), while RRBS offers a base-resolution analysis of CpG-rich regions through the combined use of enzymatic digestion (MspI) and the size selection of restriction fragments. Because no data were available regarding the optimal size window for RRBS in cattle, we conducted an in silico prediction of the genome coverage by RRBS and compared the results with those of MeDIP-chip (Additional file 1: Supplementary methods, Tables S2-S3). These in silico analyses suggested that the RRBS procedure could be successfully adapted to the bovine genome using MspI with a size window of 40-290 bp, and would lead to a base-resolution map of the methylome with coverage that would complement that of the MeDIP-chip.

**Fig. 2:**
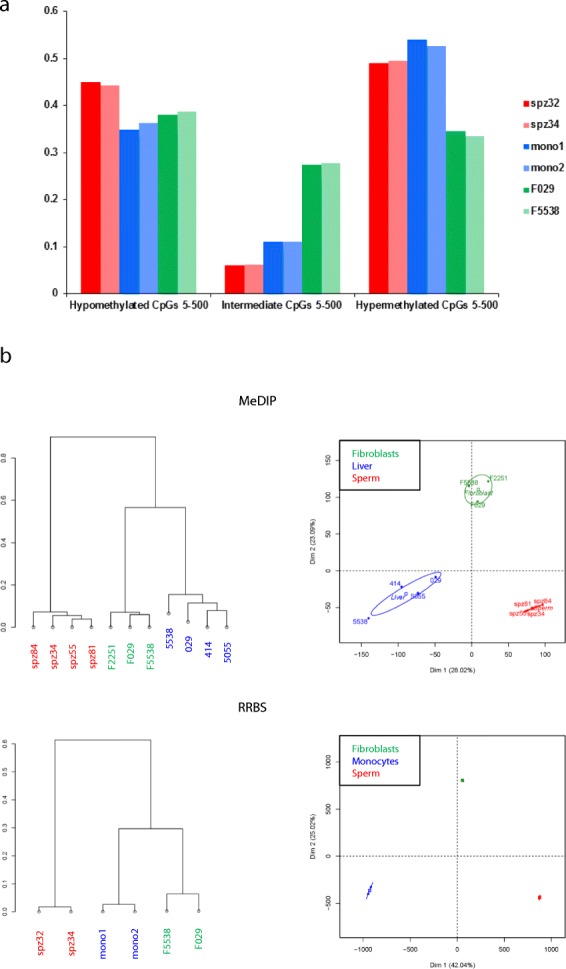
Cell type is a major determinant of DNA methylation landscapes in cattle. **a** Proportion of hypo- (< 20% methylation), intermediate (20–80% methylation) and hypermethylated CpGs (> 80% methylation) in each RRBS library, showing contrasted distributions between cell types. **b** Descriptive analyses. Upper panel: MeDIP-chip on sperm (*n* = 4, red), fibroblasts (*n* = 3, green) and liver (n = 4, blue). For each sample i and each promoter p, a normalized number of enriched probed NEpi was computed (see Additional file 1: Supplementary methods). Correlation clustering and PCA were then performed on the matrix of the normalized number of enriched probes. Lower panel: RRBS on sperm (*n* = 2, red), fibroblasts (n = 2, green) and monocytes (*n* = 2, blue). Correlation clustering and PCA were run on the totality of CpGs covered between 5 and 500 reads in the six samples

Independent bovine samples were analyzed using MeDIP-chip (sperm, liver and fibroblasts) and RRBS (sperm, monocytes and fibroblasts) in order to determine cell type-dependent variations of the methylome using these two complementary technologies. For RRBS we selected CpGs covered by 5 to 500 uniquely mapped reads for each sample (CpGs 5-500), from which an average methylation rate was calculated. The average methylation in sperm (51.8%) was higher than in fibroblasts (48%) and lower than in monocytes (57.6%; Additional file 1: Table S6). We next categorized the CpGs 5-500 into hypo- (< 20% methylation), intermediate (20–80% methylation) and hypermethylated CpGs (> 80% methylation) and observed a larger proportion of hypo- and a smaller proportion of intermediate CpGs in sperm than in somatic cells, which was counterbalanced by a large proportion of hypermethylated CpGs (Fig. 2a). This bimodal distribution of methylation in sperm probably explained the intermediate level of average methylation for CpGs 5-500. It was noted that when only CpGs covered by more than 500 uniquely mapped reads were considered (CpGs > 500), average methylation increased in monocytes (81.4%) and in fibroblasts (66.6%), but fell dramatically in sperm (22.5%; Additional file 1: Table S6).

We next conducted descriptive analyses of the MeDIP-chip and RRBS data. For MeDIP-chip, a normalized factor NEpi, representing the number of enriched probes at promoter p for sample i, was calculated for all promoters and samples, and hierarchical clustering and principal component analysis (PCA) were run on the resulting matrix. For RRBS, PCA and hierarchical clustering were computed from the matrix of methylation percentages obtained for each CpG 5-500 and each sample (Fig. 2b). With both types of descriptive analysis, the samples were clearly clustered according to the tissue/cell type using both MeDIP-chip and RRBS. Interestingly, hierarchical clustering revealed that the distance between sperm and other cell types was more important than the distance between liver and fibroblasts or monocytes and fibroblasts, highlighting the methylation specificities of germinal cells compared to somatic cells. This could also be seen in PCA, where dimension 1 opposed sperm to one or both somatic cells/tissue, while the difference between the two somatic cell types was more apparent along dimension 2.

Our results therefore showed that the tissue/cell type represented the main source of variation in methylation, and that sperm-specific methylation profiles could emerge from our MeDIP and RRBS data.

### Identification of regions and CpGs hypomethylated in bull sperm

We next ran a differential analysis on each pair of tissue/cell types. For MeDIP, we compared the proportion of enriched probes between two tissues/cell types in 27,684 regions of interest containing clusters of probes enriched in at least one tissue/cell type (see Additional file 1: Supplementary methods). This led to the identification of 4329 DMRs between sperm and liver; 3780 DMRs between sperm and fibroblasts and 2803 DMRs between fibroblasts and liver. The features of each DMR and the corresponding promoter regions are summarized in Additional file 3. For RRBS, we identified 298,901 DMCs between monocytes and sperm; 450,971 DMCs between fibroblasts and sperm, and 239,036 DMCs between monocytes and fibroblasts using stringent criteria (Additional file 1: Supplementary methods, Table S7 and Additional file 2).

**Fig. 3:**
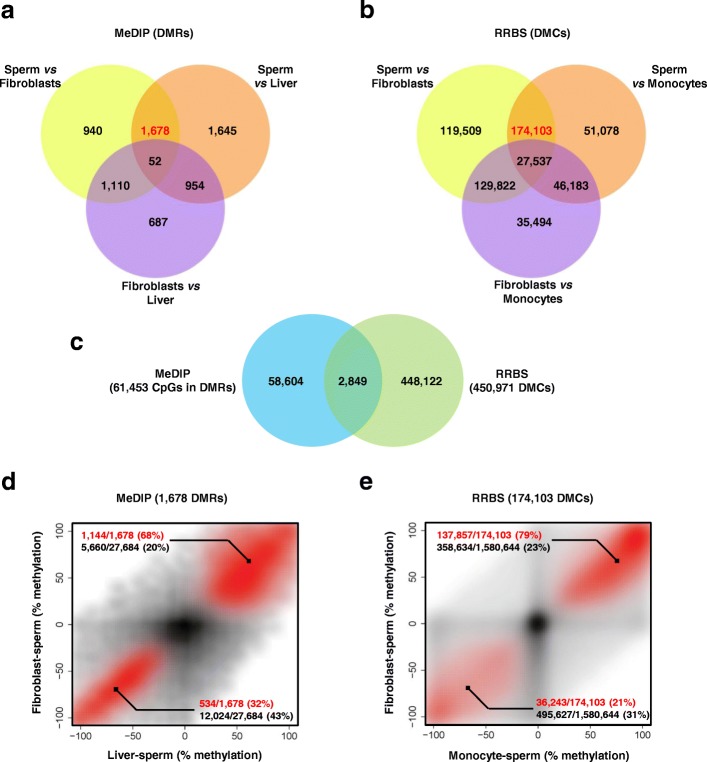
Two distinct sets of differentially methylated regions and differentially methylated CpGs display undermethylation in sperm. **a** Venn diagram showing the DMRs identified using MeDIP-chip in sperm, fibroblasts and liver. A total of 1678 DMRs specific to the comparison between sperm and somatic cells was obtained (in red). **b** Venn diagram showing the DMCs identified using RRBS in sperm, fibroblasts and monocytes. A total of 174,103 DMCs specific to the comparison between sperm and somatic cells was obtained (in red). **c** The CpG positions included in the 3780 DMRs identified between sperm and fibroblasts using MeDIP were extracted. The Venn diagram shows the intersection between these CpGs and the 450,971 DMCs identified between sperm and fibroblasts using RRBS. **d**, **e** Scatterplots showing the methylation differences between two somatic tissues and sperm, for all regions or CpGs used during differential analysis (in black; background) and for DMRs/DMCs specific to the comparison between sperm and somatic cells (in red). The proportions of background regions/CpGs overmethylated (upper right edge) and undermethylated (lower left edge) in both somatic tissues compared to sperm are indicated in black. The proportions of DMRs/DMCs overmethylated and undermethylated in both somatic tissues compared to sperm are indicated in red. **d** Differences in methylation between liver and sperm (Pr_liver_ - Pr_sperm_; x-axis) and between fibroblasts and sperm (Pr_fibroblasts_ - Pr_sperm_; y-axis) for regions and DMRs identified using MeDIP (see Additional file 1: Supplementary methods for the definition of Pr_sperm_, Pr_liver_ and Pr_fibroblasts_). **e** Differences in methylation between monocytes and sperm (x-axis) and between fibroblasts and sperm (y-axis) for CpGs and DMCs identified using RRBS

Consistent with the results of clustering and PCA, the number of DMRs/DMCs was higher between sperm and any somatic cell type/tissue than between two somatic cell types, for both MeDIP and RRBS. Fig. 3a-b shows the distribution of DMRs and DMCs in different Venn territories corresponding to pairwise comparisons under MeDIP (Fig. 3a) and RRBS (Fig. 3b). DMRs and DMCs specific to the comparison between sperm and somatic cells could be deduced from these territories (in red). These 1678 DMRs and 174,103 DMCs were located at the intersection between the “sperm *vs.* somatic cell type 1” and “sperm *vs*. somatic cell type 2” territories (yellow and orange) excluding the DMRs/DMCs also shared by the “somatic cell type 1 *vs*. somatic cell type 2” comparison (three-color territory). Because the comparison between sperm and fibroblasts was performed using both MeDIP and RRBS, the actual complementarity of the two technologies could be assessed using real data. As shown in Fig. 3c, only a limited subset of CpGs was shared by DMRs identified using MeDIP and DMCs identified using RRBS. This result, together with the in silico analysis indicating that the targeted regions were largely different under MeDIP-chip and RRBS, clearly demonstrated that we were able to identify two distinct subsets of sperm-specific DMRs/DMCs.

We next investigated whether these two subsets of sperm-specific DMRs/DMCs might display similar features in terms of variations in methylation. For both MeDIP (Fig. 3d) and RRBS (Fig. 3e), we plotted the differences in methylation between each somatic cell type and sperm for sperm-specific DMRs/DMCs. The appropriate background was used for each technology: the 27,684 regions of interest subjected to differential analysis in MeDIP and the 1,580,644 CpGs 5-500 covered in all six samples analyzed using RRBS. With both MeDIP and RRBS, background regions/CpGs (in black) were particularly concentrated around the center of the plot, illustrating that most of the regions/CpGs analyzed did not display cell type-dependent variations. By contrast, sperm-specific DMRs/DMCs were located along a diagonal that ran from the lower left-hand corner to the upper right-hand corner, meaning that these DMRs/DMCs behaved similarly in the two somatic cell types when compared to sperm. Most strikingly, a great majority of sperm-specific DMRs/DMCs (68% for MeDIP and 79% for RRBS) were grouped in the upper right-hand corner, indicating that they were hypomethylated in sperm when compared to the two somatic cell types examined.

Taken together, these results demonstrated that using two complementary technologies we were able to identify two distinct subsets of DMRs/DMCs specific to the comparison between sperm and somatic cells, most of them being less methylated in sperm. These hypomethylated sperm-specific DMRs/DMCs (hypo-DMRs/DMCs) could therefore partly explain the lower global DNA methylation of sperm observed in LUMA which was particularly marked in cattle.

### Hypomethylation in bull sperm targets specific genomic features and functions

**Fig. 4:**
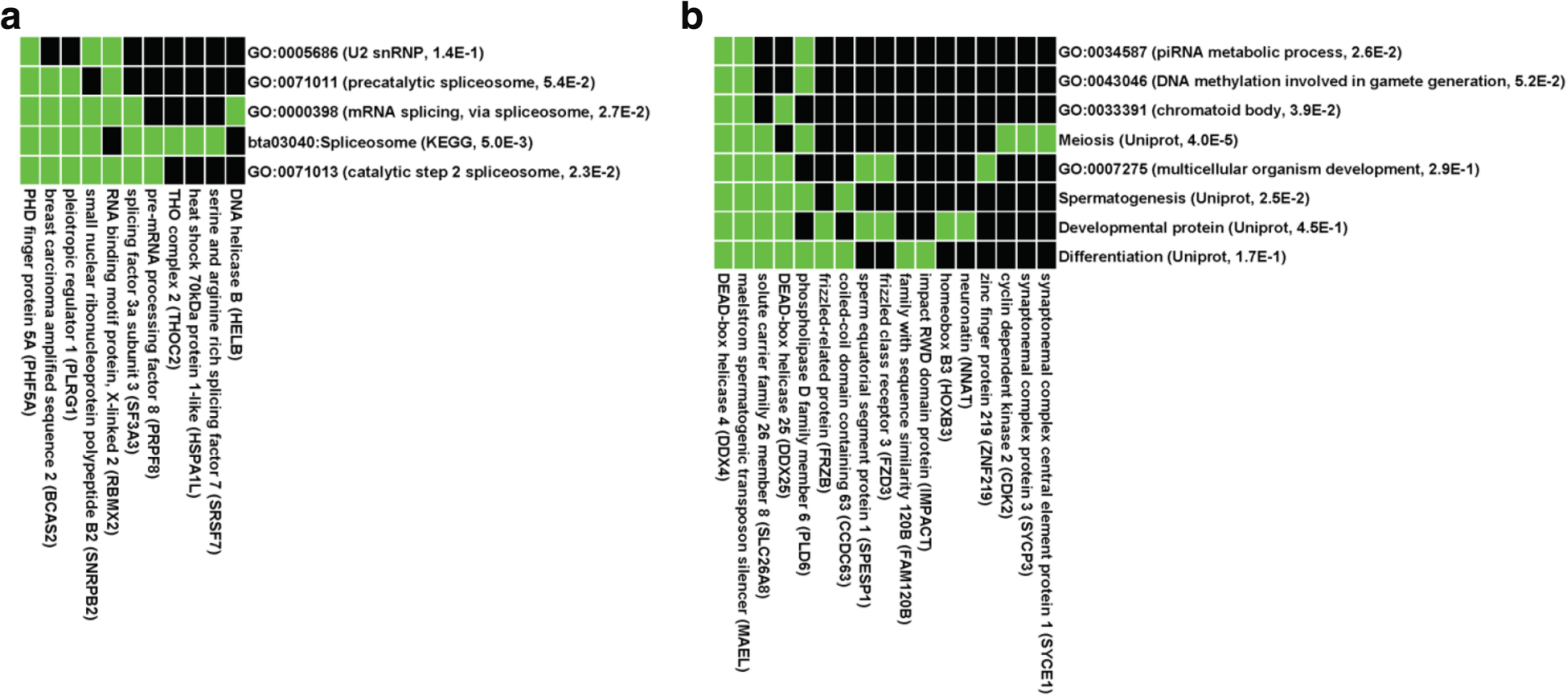
Hypo-DMRs identified by MeDIP-chip target genes involved in mRNA processing and spermatogenesis. Genes containing the 1144 hypo-DMRs were subjected to DAVID analysis, with the regions of interest used as the background. Terms of gene ontology, pathways or Uniprot keywords enriched among the DMRs and their corresponding p-values are indicated, as are the genes present in each category. The green color on the heatmap represents a correspondence between a gene and a category. To limit the size of the heatmaps, only GO terms designated as DIRECT by DAVID were used for cluster generation. **a** Functional cluster related to mRNA processing. **b** Functional cluster related to meiosis and spermatogenesis

To determine whether specific gene ontology (GO) terms were enriched in the sperm hypomethylated regions, we next annotated the hypo-DMRs identified by MeDIP relative to genes. Consistent with the microarray design, most of the 1144 hypo-DMRs were located in or close to genes according to the criteria described in the Methods, resulting in a list of 701 unique genes which were then subjected to DAVID analysis. Significant enrichments were found for biological processes such as sexual reproduction (36 genes), fertilization (15 genes) and RNA transport (11 genes). Further analysis using a more restrictive list of GO terms led to the identification of functional clusters involved in mRNA processing (Fig. 4a) and meiosis/spermatogenesis (Fig. 4b).

**Fig. 5:**
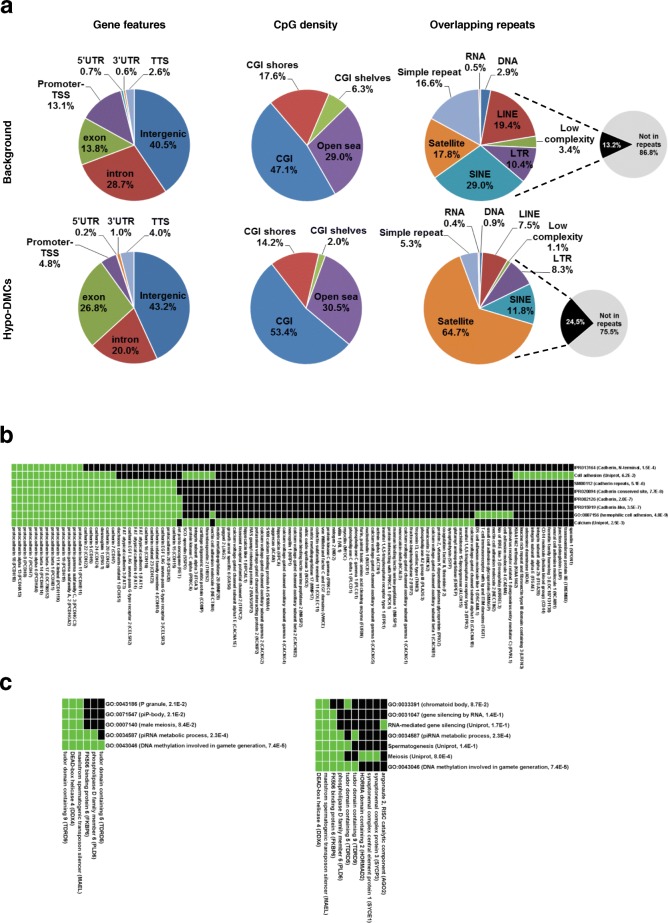
Hypo-DMCs identified by RRBS target specific genomic features and functions. The 137,861 hypo-DMCs and 1,580,644 CpGs (background) were annotated relative to gene features, CpG density and overlapping repeats. **a** Distribution of hypo-DMCs and background CpGs among these genomic features. **b** Genes with hypo-DMCs located in exons were subjected to DAVID analysis, with genes from some of the 1,580,644 CpGs in exons used as the background. The heatmap represents a functional cluster related to cell adhesion. **c** Genes with hypo-DMCs located in promoter-TSS were subjected to DAVID analysis, with genes containing some of the 1,580,644 CpGs in promoter-TSS used as the background. The heatmaps represent functional clusters related to piRNA metabolism (left panel) and to meiosis and spermatogenesis (right panel). To limit the size of the heatmaps, only GO terms designated as DIRECT by DAVID were used for cluster generation

Because CpGs targeted by RRBS are scattered along the genome, we then started to characterize the hypo-DMCs identified by RRBS relative to different genomic features (genes, CpG density and overlapping repeats; Fig. 5a). The most remarkable observation was a dramatic enrichment of hypo-DMCs for repeats (24.5% vs. 13.2% in background; right panel), and particularly for satellites (64.7% vs. 17.8%). In order to get a more precise picture of the methylation status of repetitive elements by rescuing some of the information included in the ambiguous reads, we aligned the reads on a Repbase artificial genome containing the consensus sequence of each bovine repeat (see Additional file 1: Supplementary methods for details, and Additional file 4 and Additional file 1: Table S8 for data). The hypomethylation of sperm was clear in satellites and also in rDNA repeats encoding ribosomal RNAs (Additional file 1: Figure S3). We therefore concluded from both CpGs targeted by uniquely mapped reads (that had been used to identify hypo-DMC) and CpGs targeted by ambiguous reads, that specific families of repetitive elements were massively hypomethylated in bovine sperm.

Regarding gene features with respect to RRBS data, the proportion of genes containing hypo-DMCs was relatively unchanged compared with background (Fig. 5a, left panel), but interestingly, exons were more frequently represented (26.8% vs. 13.8% in background) while promoter-TSS were represented less than in background (4.8% vs. 13.1%). The distribution of hypo-DMCs in CGIs, shores and shelves was identical to that observed with the background (middle panel). We investigated whether specific GO terms were enriched in gene features displaying a different representation in hypo-DMCs and background. For exonic hypo-DMCs, which accounted for 2713 unique genes, significant enrichments were found for biological processes such as cell adhesion (213 genes), the regulation of signaling (382 genes) and cell migration (177 genes), and one main functional cluster related to cell adhesion could also be identified (Fig. 5b). For hypo-DMCs located in promoter-TSS, which accounted for 1200 unique genes, the most enriched biological process was sexual reproduction (44 genes), and two functional clusters were identified (Fig. 5c) as being related to piRNA metabolism (left panel) and to meiosis and spermatogenesis (right panel).

From an analysis of both hypo-DMRs obtained in MeDIP and hypo-DMCs identified through RRBS, we therefore concluded that undermethylation in sperm essentially targeted repeats and the promoters of genes important to spermatogenesis (which is the differentiation process that eventually leads to the mature sperm we analyzed), but also to genes involved in cell communication, signaling and migration that may be essential to both sperm functions and post-fertilization steps.

### Hypomethylation of four regions is confirmed by bisulfite-pyrosequencing

**Fig. 6:**
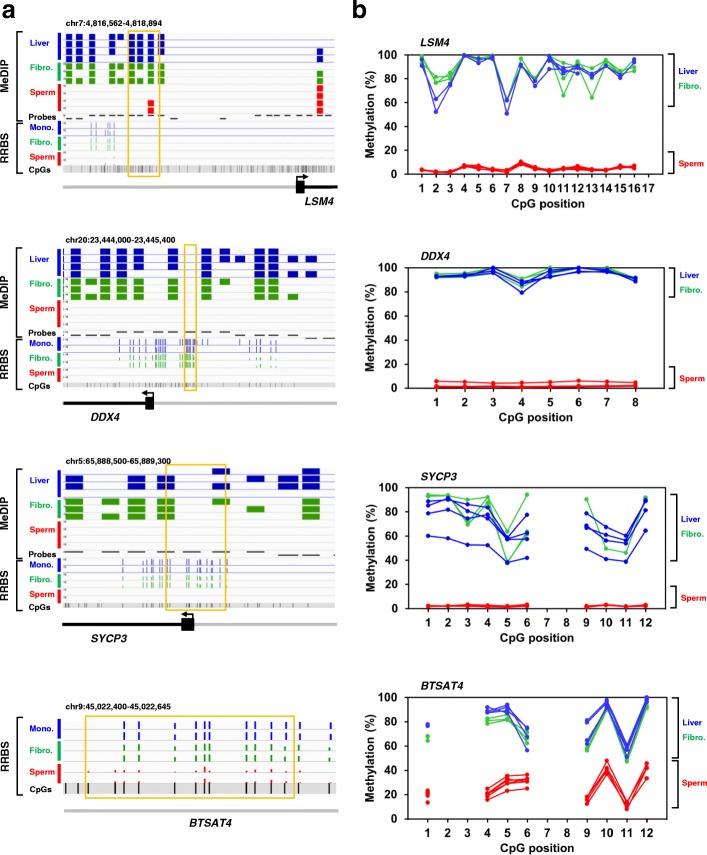
Validation by bisulfite-pyrosequencing. **a** IGV browser views of the gene regions targeted for pyrosequencing. In the MeDIP-chip panels, the “Probes” track indicates the probe positions on the microarray. The blue, green, and red bar charts represent probes with signal enrichment in the MeDIP fraction for liver, fibroblast and sperm samples, respectively. An absence of chart at a given probe position denotes that signal was not enriched in the MeDIP fraction. In the RRBS panels, the blue, green, and red bar charts represent the methylation percentages at each CpG 5-500 position for monocyte, fibroblast and sperm samples, respectively. Individual CpGs are shown, as are the MeDIP probe classes based on CpG frequency (the upper, middle and lower bands represent high, intermediate and low class probes, respectively). The orange boxes delineate the regions analyzed by pyrosequencing. **b** Methylation percentages of the CpGs assayed by pyrosequencing in sperm (*n* = 6), fibroblasts (*n* = 3) and liver (*n* = 4). The difference between sperm and somatic cells is significant at every position (*p* < 0.05, permutation test)

Four regions were selected for validation, based on their hypomethylation in sperm, the presence of both DMRs obtained by MeDIP analysis and DMCs obtained by RRBS analysis whenever possible, and their position relative to genes involved in sperm functions. Figure 6a shows the detailed localization of these regions together with their coverage and individual methylation in both MeDIP and RRBS. *LSM4*, which contained a hypo-DMR, is a gene involved in RNA processing [45]. Genes *SYCP3* and *DDX4,* which contained both hypo-DMRs and hypo-DMCs identified in our study, play a major role in spermatogenesis insofar as either mutation or aberrant methylation of these genes associate to male infertility [46, 47]. *BTSAT4* was the most frequently represented bovine satellite in our RRBS data and displayed undermethylation in sperm (Additional file 4). Overall, the four regions represented 42 analyzed CpGs. We used the pyrosequencing of bisulfite-converted DNA [40] to quantify the absolute methylation percentage of individual CpGs in sperm, liver and fibroblasts (Fig. 6b; genomic DNA from monocytes was in limited amounts and was saved for separate investigations). Consistent with the MeDIP data showing enriched probes along the *LSM4* promoter in liver and fibroblasts but not in sperm, the CpGs analyzed by pyrosequencing were all hypomethylated in sperm. In this region, the CpGs assessed by pyrosequencing were not covered by RRBS, but those in *DDX4*, *SYCP3* and *BTSAT4* were covered by the three techniques and displayed hypomethylation in sperm whatever the technique used. By pyrosequencing four additional CpGs, we also checked that one region specifically hypermethylated in sperm compared to somatic cells validated (Additional file 1: Figure S4).

In conclusion, the results obtained using MeDIP, RRBS and pyrosequencing were in excellent agreement, which validated the high-throughput data and led to the characterization of gene regions with methylation patterns specific to bull sperm.

## Discussion

During this study, we performed DNA methylation analyses at different genome scales in order to exhaustively characterize the bovine sperm methylome. Our main findings were that global DNA methylation level was low in bull sperm compared with other species, and that bull sperm was less methylated than bovine somatic cells in the context of two genome-wide methylation assays targeting distinct genomic regions, namely RRBS and MeDIP-chip, with a focus on gene promoters.

The undermethylation of bull sperm compared to somatic cells agreed well with findings in other mammals and provides additional evidence of features of the male germline differentiation program being conserved across species. Hypomethylated loci have been identified for instance in the sperm of human and chimp using whole genome bisulfite sequencing [48] or the Infinium 450 K methylation platform [19, 49]. Together with our work, these reports demonstrate that the undermethylated status of sperm is independent of both the genome coverage and resolution of the technology used to map DNA methylation, and suggest that the presence of hypomethylated loci is conserved among mammals. We found that promoters and exons of genes hypomethylated in bull sperm were enriched for biological processes essential to sperm functions, such as sexual reproduction, fertilization, cell adhesion and migration, meiosis, RNA transport and processing (including piRNA metabolism), and the regulation of signaling. Interestingly, genes involved in these processes displayed highly dynamic expression in post-natal mouse spermatogonial stem cells [50]. In human sperm, some of these processes (cell adhesion, sexual reproduction, meiosis and piRNA metabolism) are enriched in hypomethylated promoters [48]. Hypermethylation of the piRNA machinery in testes has also been associated with human spermatogenic disorders [51]. In several species including bovine, the undermethylation of mature spermatozoa could therefore reflect a dynamic sequence of past transcriptional events in the male germline differentiation program which are essential to sperm functions.

Another striking finding revealed by our RRBS data was the undermethylation of repetitive elements in bull sperm, especially satellites. How to analyze repetitive sequences is still a matter of debate because of potential mapping artefacts [52, 53]. We initially decided to discard ambiguous reads, which may have led to an underestimation of the total contribution of repeats to hypomethylated loci in sperm. Alternatively, we aligned the sequences on an artificial genome that contained one copy of each bovine repetitive element, and were able to confirm the undermethylation of satellites and rDNA repeats encoding ribosomal RNAs in bull sperm. The undermethylation of satellites in sperm has long been described in several species, including bovine, by analyses of candidate sequences [54–56]. More recently, the undermethylated status of satellites in human and chimp sperm has been generalized to the whole genome [48]. Satellites are essential components of the constitutive heterochromatin in mammals, and this function is partly mediated by DNA methylation [57]. Satellites play key roles in chromosome structure, stability and segregation. Through their high molecular dynamics and ability to drive chromosome rearrangements, they are considered to be major actors in diseases such as cancer, but also in genome evolution and speciation [58]. The significance of satellite undermethylation in sperm could be related to the transcriptional burst that arises from paternal satellites in early mouse development, which is necessary for normal formation of the heterochromatin in embryos and for developmental progression [59]. Consistent with this important transcriptional activity, satellites remain hypomethylated after fertilization in normal preimplantation embryos [56, 60]. In contrast, embryos resulting from somatic cell nuclear transfer (SCNT) and have a reduced development potential, display somatic-like hypermethylated satellites in the mouse [56] and bovine [61–63]. Of note, the hypermethylation of satellites seems to persist in the sperm of adult SCNT-derived bulls [64], suggesting that it has resisted the two waves of epigenetic reprogramming that occur during early development and germ cell differentiation.

Strikingly, the sperm-specific hypomethylated sequences identified throughout this study, and which were particularly enriched in genes related to germline differentiation and in satellite and rDNA repeats, displayed several common features. Firstly, these hypomethylated sequences have previously been described as targets of *DNMT3B* de novo DNA methyltransferase. Indeed, the methylation of satellites is half-reduced in the germline of newborn male mice deficient for *DNMT3B* [65]. In humans, *DNMT3B* mutations lead to the ICF syndrome (Immunodeficiency Centromeric instability Facial anomalies). In somatic cells, this disease is associated with a hypomethylated status of germline genes and centromeric satellites that closely mimics that of gametes, affecting nuclear organization and chromosome stability [66]. Another common feature shared by sperm-specific hypomethylated sequences is that they partly remain associated to nucleosomes in mature spermatozoa, as reported for genes involved in RNA processing and for repetitive elements (including centromeric satellites) in humans and bovine [67], and for satellites and rDNA repeats in bovine [68]. In addition, sperm histone retention particularly affects CpGs that lack methylation in humans [49] and the mouse [69]. The association with retained nucleosomes in sperm, together with the important function of paternal satellite transcripts in early embryos and the hypermethylation of satellites in SCNT embryos, support the hypothesis that regions which are hypomethylated in sperm play a fundamental role not only in germline differentiation but also in post-fertilization epigenetic reprogramming.

The final question arising from our study originates from the intriguing finding that global methylation at CCGG sites was more than 10% lower in bull sperm than in any other species investigated (sheep, horse, pig, mouse, goat and human), which remains to be confirmed using larger samples. Because satellites are undermethylated in the sperm of many species, the global undermethylation of bull sperm could partly be explained by the larger amount of satellite sequences present in the bovine genome (eight satellite components representing 23% of the total genomic content; [70]). We annotated the CCGG sites relative to the genomic features available in different species, and indeed observed that CCGGs overlapping satellites represented 1.6% of all CCGGs in bovine vs. 0.1 to 0.6% in other species. From a purely mathematical point of view, the low percentage of CCGG sites present in satellites whatever the species probably did not accounted for the > 10% difference in sperm methylation. However, it should be kept in mind that the repetitive nature of satellites precludes their correct integration in genome assemblies, which probably led to an underestimation of their contribution to global CCGG methylation. Although we cannot rule out that the weaker global methylation in bull sperm is due to a higher representation of bovine satellites in CCGGs, an alternative explanation might be that satellite methylation is quantitatively lower in bull sperm than in the sperm of other species. This is supported by an old report which demonstrated that relative to somatic tissues, most satellites are largely undermethylated in the sperm of cattle while they are only slightly undermethylated in mouse sperm [54]. The abundance of satellites in the bovine genome, together with their low methylation content in male germ cells, may contribute to explaining some of the bovine-specific features of meiotic recombination. Crossing-over events are more frequently observed in the spermatocytes of *Bos taurus* than in those of related Bovidae species (wildebeests; [71]). Moreover, the meiotic recombination rate in cattle is particularly elevated in males, while in most species it is higher in females [72, 73]. The frequency of crossing-overs is usually low in repeat-rich domains associated with heterochromatin [74]; however, the weak methylation of satellites in bull germ cells probably reflects a particular chromatin structure that may promote crossing-overs. The undermethylation of bull spermatozoa may also have contributed to shaping the bovine genome. Indeed, segmental duplications in the bovine reference genome are particularly enriched for satellite repeats that are undermethylated in bull sperm, including *BTSAT4* [75]. Segmental duplications promote both chromosome rearrangements that drive bovine genome evolution (as indicated by their enrichment in the vicinity of cattle-specific evolutionary breakpoints [29]), and inter-individual variability through their ability to promote CNVs [76]. The role of segmental duplications in structural variations of the bovine genome might be mediated by hypomethylated sequences in the bull germline. In support of this hypothesis, human-specific evolutionary rearrangements and CNVs associate not only with low copy repeats and the deletions/duplications they generate, but also with hypomethylated regions of human sperm [77], thus providing a potential link between hypomethylation in the germline and genome structural variation.

## Conclusions

By means of a thorough characterization of bull sperm DNA methylation at different genome scales, this study has provided evidence that bull spermatozoa are less methylated when compared to not only bovine somatic cells but also the sperm of other mammals. The sequences undermethylated in bull sperm are conserved across species, which may denote an important role in germline differentiation and in post-fertilization epigenetic reprogramming. The cattle-specific lower methylation at CCGG sites may be partly related to the abundance of satellites in the bovine genome and to their undermethylated status in the male germline. Given the potential evolutionary implications of these findings, it would be of considerable interest to quantify DNA methylation at different stages of bovine male germline differentiation in order to understand when and how this undermethylation takes place.

## Additional files

Additional file 1: is a pdf file containing supplementary methods, supplementary references, eight supplementary tables and four supplementary figures. **Table S1.** reference genomes used for in silico analyses and origin of the files used for annotation. **Table S2.** in silico characterization of bovine reduced restriction (RR) genomes generated using different size selection criteria. **Table S3.** comparison of RR genomes obtained with a 40-290 bp selection size window in different species. **Table S4.** primers and PCR conditions used to generate the pyrosequencing templates. **Table S5.** pyrosequencing primers. **Table S6.** library characterization, mapping efficiency on the bovine genome (UMD3.1), coverage and average methylation in RRBS libraries. **Table S7.** results of comparisons between tissues by RRBS. **Table S8.** mapping efficiency on a Repbase artificial bovine genome, coverage and average methylation in RRBS libraries. **Figure S1.** Bootstrap analysis of global CCGG methylation in bull sperm from four different breeds. **Figure S2.** Genotyping of bull sperm and blood samples. **Figure S3.** Average methylation percentages for CpGs 5-500 and CpGs > 500 in each cell type, in reads uniquely aligned on a Repbase bovine artificial genome. **Figure S4.** Pyrosequencing of CpGs hypermethylated in sperm. (PDF 1840 kb)

Additional file 2: is a Microsoft Excel file listing the DMCs identified using RRBS. This file includes three datasheets corresponding to the pairwise comparisons between sperm, fibroblasts and monocytes. (XLSX 84756 kb)

Additional file 3: is a Microsoft Excel file listing the DMRs identified using MeDIP. This file includes three datasheets corresponding to the pairwise comparisons between sperm, fibroblasts and liver. One DMR may be present in several lanes if shared by several promoters. (XLSX 821 kb)

Additional file 4: is a Microsoft Excel file listing the consensus sequences of each bovine repetitive element as defined in Repbase and the average methylation percentages for CpGs 5-500 and CpGs > 500 in each RRBS sample. (XLSX 16 kb)

## Abbreviations

CGI: CpG island
CNV: Copy number variation
DAVID: Database for Annotation, Visualization and Integrated Discovery
DMC: Differentially methylated CpG
DMR: Differentially methylated region
DTT: Dithiothreitol
GO: Gene ontology
IGV: Integrative Genomics Viewer
LUMA: Luminometric methylation assay
MeDIP: Methylated DNA immunoprecipitation
PBMCs: Peripheral blood mononuclear cells
PBS: Phosphate buffer saline
PCA: Principal component analysis
RR genome: Reduced representation genome
RRBS: Reduced representation bisulfite sequencing
SCNT: Somatic cell nuclear transfer
TSS: Transcription start site
TTS: Transcription termination site
