# Supplementary material for: A multi-scale analysis of bull sperm methylome revealed both species peculiarities and conserved tissue-specific features
Source: BMC Genomics. 2018 May 29;19:404. doi: 10.1186/s12864-018-4764-0 (PMC5975405; doi:10.1186/s12864-018-4764-0)
Supplement: Supplementary file 1 — is a pdf file containing supplementary methods, supplementary references, eight supplementary tables and four supplementary figures. Table S1. reference genomes used for in silico analyses and origin of the files used for annotation. Table S2. in silico characterization of bovine reduced restriction (RR) genomes generated using different size selection criteria. Table S3. comparison of RR genomes obtained with a 40-290 bp selection size window in different species. Table S4. primers and PCR conditions used to generate the pyrosequencing templates. Table S5. pyrosequencing primers. Table S6. library characterization, mapping efficiency on the bovine genome (UMD3.1), coverage and average methylation in RRBS libraries. Table S7. results of comparisons between tissues by RRBS. Table S8. mapping efficiency on a Repbase artificial bovine genome, coverage and average methylation in RRBS libraries. Figure S1. Bootstrap analysis of global CCGG methylation in bull sperm from four different breeds. Figure S2. Genotyping of bull sperm and blood samples. Figure S3. Average methylation percentages for CpGs 5-500 and CpGs > 500 in each cell type, in reads uniquely aligned on a Repbase bovine artificial genome. Figure S4. Pyrosequencing of CpGs hypermethylated in sperm. (PDF 1840 kb) [file 12864_2018_4764_MOESM1_ESM.pdf]

Supplementary information

A multi-scale analysis of bull sperm methylome revealed both species peculiarities and conserved tissue-specific features

Jean-Philippe Perrier[1,2], Eli Sellem[1,3], Audrey Prézelin[1], Maxime Gasselin[1], Luc Jouneau[1], François Piumi[1,4,5], Hala Al Adhami[1,6], Michaël Weber[6], Sébastien Fritz[3,7], Didier Boichard[7], Chrystelle Le Danvic[3,8], Laurent Schibler[3], Hélène Jammes[1] and Hélène Kiefer[1]*

[1]UMR BDR, INRA, ENVA, Université Paris Saclay, 78350, Jouy en Josas, France

[2]Present Address: Laboratory of Animal Reproduction, Department of Biological Sciences, Faculty of Science and Engineering, University of Limerick, Limerick, Ireland

[3]ALLICE, 149 rue de Bercy, 75012, Paris, France

[4]Present Address: Institut Curie, PSL Research University, CNRS, UMR3664, 75005, Paris, France

[5]Present Address: Sorbonne Universités, UPMC Univ Paris 06, CNRS, UMR3664, 75005, Paris, France

[6]CNRS, Université de Strasbourg, UMR7242 Biotechnologie et signalisation cellulaire, 300 bd Sébastien Brant, 67412 Illkirch cedex, France

[7]UMR GABI, INRA, AgroParisTech, Université Paris Saclay, 78350, Jouy en Josas, France

[8]UMR CNRS/USTL 8576, UGSF, Villeneuve D'Ascq, France

*Corresponding author

Supplementary methods

MeDIP-chip data analysis

Probes with signal enrichment in the MeDIP sample ("enriched probes") were identified using the ChIPmix R package [1] and could be visualized using Integrative Genomics Viewer software (IGV) [2]. For Fig. 2b, a normalized enrichment factor NEpi was calculated for each promoter p and each individual i as follows:

$\mathrm{NEpi} = \mathrm{Epi}\frac{\mathrm{G}}{\mathrm{Gi}}$, where:

Epi =Number of enriched probes for promoter p and sample i;

$\mathrm{Gi} = \sum_{\mathrm{p}=1}^{21296} \mathrm{Epi}$ = Total number of enriched probes at promoters for sample i;

$\mathrm{G} = \frac{1}{11}\sum_{\mathrm{i}=1}^{11}\sum_{\mathrm{p}=1}^{21296} \mathrm{Epi}$ =Mean Gi for all samples.

Principal component analysis (PCA) and hierarchical clustering were then computed on the NEpi matrix using the FactoMineR R package. For hierarchical clustering, the distance between samples was calculated using Pearson correlation coefficients and Ward's method was applied as the linkage function.

To identify regions of interest containing clusters of probes enriched in at least one tissue, each promoter was screened for an anchor probe displaying enrichment in at least two samples from the same tissue. Starting from this anchor, the region was then extended to upstream and downstream probes until a probe displaying less than 10% enrichment in any tissue was encountered. Two consecutive regions of interest separated by fewer than three probes were coalesced into one larger region of interest. After coalescence, regions containing only the anchor probe were eliminated. Indeed, the signal from a single probe could result from cross-hybridization with another genomic region, whereas this probability might be reduced if several probes were locally enriched. This strategy led to the identification of 27,684 regions of interest.

Among these regions of interest, those displaying differential methylation between tissues were identified using an R package designed to analyze point patterns [3] which was therefore appropriate for binary data (enriched *vs.* not enriched). For each pair of tissues, two models were built and estimated using the ppm function of the spatstat R package (ANOVA for fitted point process models): the full model took account of the origin of the tissue while the alternative model did not. To identify differentially methylated regions (DMRs), the full model was compared with the alternative model. All 27,684 regions of interest were tested and the resulting p-values were corrected for multiple testing using the Benjamini-Hochberg procedure [4] with the multitest R package. A given region was considered to be a DMR if the full model fitted the observations significantly better than the alternative model (adjusted p-value <0.05).

The mean percentage of enriched probes Pr in each tissue was calculated as follows for each region r (DMR or region of interest):

$\mathrm{Pr} = \frac{1}{\mathrm{n}}\sum_{\mathrm{i}=1}^{\mathrm{n}}\frac{\mathrm{Eri}}{\mathrm{Tr}} \times 100$, where n is the total number of biological replicates in the tissue considered (n=4 for liver and sperm, n=3 for fibroblasts), Eri is the number of enriched probes for region r and biological replicate i, and Tr is the number of probes included in region r.

Reduced representation genome *in silico* analysis

We produced five reduced representation (RR) genomes digested *in silico* by MspI and displaying size windows of 250 bp with a 40 bp increment (from 0-250 bp to 160-410 bp). The results are summarized in Additional file 1: Table S2. Consistent with the microarray design, the coverage in terms of promoter-TSS and genes was higher for the MeDIP-chip than for any RR genome, illustrating the complementarity of these two approaches. While the percentage of the whole genome covered in RR genomes increased from 2.7% to 3.2% with size window increments, the CpG sites covered decreased from 13% to 9.7%. This decrease paralleled the fall in CGI and intragenic percentages. The 0-250 bp RR genome was the most cost-effective in terms of the CpGs covered versus the fraction of

genome sequenced. This RR genome was also interesting in terms of the gene features covered, with the highest percentage of CpGs included in genes, promoter-TSS, exons and CGIs, and the lowest percentage of CpGs included in repeats. However, we selected the 40-290 bp RR genome because very short fragments have a high probability of mapping on multiple locations [49, 50] and are little informative after sequencing. This RR genome was also close to the 40-220 bp size window which is widely used in other species [38, 49, 51]. We then compared the bovine 40-290 bp RR genome with 40-290 bp RR genomes from other species, and noticed that a roughly similar CpG enrichment ratio was obtained in all species (ratio between the number of CpGs per Mb RR genome and the number of CpGs per Mb whole genome; Additional file 1: Table S3).

RRBS data analysis

RRBS libraries were generated using the conditions defined above (MspI, 40-290 bp fragments). The sequences displayed the expected nucleotide composition based on MspI digestion and bisulfite conversion according to FastQC quality control (https://www.bioinformatics.babraham.ac.uk/projects/fastqc/). Subsequent quality checks and trimming were carried out using Trim Galore v0.3.7 (http://www.bioinformatics.babraham.ac.uk/projects/trim_galore/) which removed adapter sequences, poor quality bases and reads (Phred score below 20) and reads shorter than 20 nucleotides. High quality reads were aligned on the bovine reference genome (UMD 3.1 assembly) using Bismark v0.14.3 in the default mode with Bowtie 1 [5, 6]. Thirty million pairs of reads per RRBS library were obtained on average, among which 89% could be aligned to the reference genome with 36.6% unique mapping and 52.4% multi-mapping (ambiguous reads; Additional file 1: Table S6). The rate of unique mapping was lower than that reported for other mammals including sheep [7] and pig [8], but higher than that observed in zebrafish [9] and within the same range as another RRBS study conducted in cattle [10].

The bisulfite conversion rate was estimated from the unmethylated cytosine added *in vitro* during the end-repair step. The bisulfite conversion rate was ≥99.5% for all samples but did not reach 100%. Under these conditions, it was impossible to distinguish non-CpG methylation (which represents only 0.02% of total methylation in somatic cells [12]) from residual cytosines due to incomplete bisulfite conversion. We therefore focused our analysis exclusively on CpG methylation.

The CpGs were then selected based on their coverage by uniquely mapped reads. We conserved the CpGs covered by at least 5 reads, which is a usual threshold to identify differences between cell types [11], and separately considered the CpGs covered by >500 reads since excessive coverage might be related to a biased amplification of repetitive elements during library preparation. Only CpGs covered by 5 to 500 uniquely mapped reads for each sample (termed as CpGs 5-500) were retained for subsequent analyses. Each CpG 5-500 was assigned a methylation percentage per sample calculated from Bismark methylation calling $((\text{reads with "C"} \times 100)/(\text{reads with "C"} + \text{reads with "T"})) * 100$, which could be visualized using IGV. For Fig. 2b, PCA and hierarchical clustering were then computed on the matrix of methylation percentages for each CpG 5-500 and each sample as explained for the MeDIP data.

For each pair of tissues, differentially methylated CpGs (DMCs) were identified using methylKit [12]. A CpG 5-500 was considered as a DMC when the associated q-value was weaker than 0.001 and the methylation difference between the two tissues was at least 25% (according to the methylKit calculation mode, which takes account of the coverage per sample; see Additional file 2, column K). In parallel with this differential analysis, some CpGs 5-500 were selected based on contrasted methylation percentages in the two cell types (methylation percentage weaker than 20% for the two biological replicates in the first cell type and higher than 80% for the two biological replicates in the second cell type) and added to the DMC list in case they had not previously been detected by methylkit (“obvious DMCs”). The thresholds were set stringently for both methylKit DMCs and obvious DMCs in order to prevent the generation of false positives, since our experimental design included only two biological replicates per cell type.

Alignment on a Repbase artificial bovine genome

We suspected that only a small fraction of the repetitive elements was represented in the uniquely mapped reads, and hypothesized that more information on the repeats hypomethylated in sperm could be extracted from the ambiguous reads. We therefore built an artificial genome containing one copy of each bovine repeat as defined in the Repbase database [13], and aligned the totality of the reads on this artificial genome. Under these conditions, reads mapping at a unique location reached 24% on average, which was quite high considering the reduced size of the artificial genome (Additional file 1: Table S8). By contrast with the results obtained using the bovine reference genome (Additional file 1: Table S6), the number of CpGs >500 was not dramatically lower than that of CpGs 5-500, which was in line with the stacking of numerous reads at the same location on the artificial genome. The average methylation was the lowest in sperm, especially for CpGs >500, and the proportion of hypomethylated CpGs (average methylation <20%) was consistently the highest in sperm. Additional file 4 shows the average percentage of methylation calculated individually for each Repbase consensus repeat, from either CpGs 5-500 or CpGs >500, and Additional file 1: Figure S3 recapitulates the average percentage per family of repeats in each cell type.

Supplementary references

1. Martin-Magniette ML, Mary-Huard T, Berard C, Robin S: **ChIPmix: mixture model of regressions for two-color ChIP-chip analysis**. *Bioinformatics* 2008, **24**(16):i181-186.
2. Robinson JT, Thorvaldsdottir H, Winckler W, Guttman M, Lander ES, Getz G, Mesirov JP: **Integrative genomics viewer**. *Nature biotechnology* 2011, **29**(1):24-26.
3. Baddeley A, Turner R: **spatstat: An R Package for Analyzing Spatial Point Patterns**. *Journal of Statistical Software* 2005, **12**(6).
4. Benjamini Y, Hochberg Y: **Controlling the False Discovery Rate: A Practical and Powerful Approach to Multiple Testing**. *Journal of the Royal Statistical Society Series B (Methodological)* 1995, **57**(1):289-300.

5. Krueger F, Andrews SR: **Bismark: a flexible aligner and methylation caller for Bisulfite-Seq applications**. *Bioinformatics* 2011, **27**(11):1571-1572.
6. Langmead B, Trapnell C, Pop M, Salzberg SL: **Ultrafast and memory-efficient alignment of short DNA sequences to the human genome**. *Genome biology* 2009, **10**(3):R25.
7. Doherty R, Couldrey C: **Exploring genome wide bisulfite sequencing for DNA methylation analysis in livestock: a technical assessment**. *Frontiers in genetics* 2014, **5**:126.
8. Choi M, Lee J, Le MT, Nguyen DT, Park S, Soundrarajan N, Schachtschneider KM, Kim J, Park JK, Kim JH *et al*: **Genome-wide analysis of DNA methylation in pigs using reduced representation bisulfite sequencing**. *DNA research : an international journal for rapid publication of reports on genes and genomes* 2015, **22**(5):343-355.
9. Chatterjee A, Ozaki Y, Stockwell PA, Horsfield JA, Morison IM, Nakagawa S: **Mapping the zebrafish brain methylome using reduced representation bisulfite sequencing**. *Epigenetics* 2013, **8**(9):979-989.
10. Doherty R, Whiston R, Cormican P, Finlay EK, Couldrey C, Brady C, O'Farrelly C, Meade KG: **The CD4(+) T cell methylome contributes to a distinct CD4(+) T cell transcriptional signature in Mycobacterium bovis-infected cattle**. *Scientific reports* 2016, **6**:31014.
11. Meissner A, Mikkelsen TS, Gu H, Wernig M, Hanna J, Sivachenko A, Zhang X, Bernstein BE, Nusbaum C, Jaffe DB *et al*: **Genome-scale DNA methylation maps of pluripotent and differentiated cells**. *Nature* 2008, **454**(7205):766-770.
12. Akalin A, Kormaksson M, Li S, Garrett-Bakelman FE, Figueroa ME, Melnick A, Mason CE: **methylKit: a comprehensive R package for the analysis of genome-wide DNA methylation profiles**. *Genome biology* 2012, **13**(10):R87.
13. Bao W, Kojima KK, Kohany O: **Repbase Update, a database of repetitive elements in eukaryotic genomes**. *Mobile DNA* 2015, **6**:11.

Supplementary Tables

Table S1. Reference genomes used for *in silico* analyses and origin of the files used for annotation

Species	Reference genome	Gtf file	Biomart file	CpG island file	Repeat file
**Cattle**	UMD3.1	ftp://ftp.ensembl.org/pub/release-81/gtf/bos_taurus/Bos_taurus.UMD3.1.81.gtf.gz	http://jul2015.archive.ensembl.org/biomart/martview/ (Ensembl Genes 81, Bos taurus genes (UMD3.1))	http://hgdownload.cse.ucsc.edu/goldenPath/bosTau6/database/cpgIslandExt.txt.gz	http://hgdownload.cse.ucsc.edu/goldenPath/bosTau6/database/rmsk.txt.gz
**Sheep**	Oar_v3.1	ftp://ftp.ensembl.org/pub/release-87/gtf/ovis_aries/Ovis_aries.Oar_v3.1.87.gtf.gz	http://dec2016.archive.ensembl.org/biomart/martview/ (Ensembl Genes 87, Sheep genes (Oar_v3.1))	http://hgdownload.soe.ucsc.edu/goldenPath/oviAri3/database/cpgIslandExt.txt.gz	http://hgdownload.soe.ucsc.edu/goldenPath/oviAri3/database/rmsk.txt.gz
**Horse**	EquCab2	ftp://ftp.ensembl.org/pub/release-87/gtf/equus_caballus/Equus_caballus.EquCab2.87.gtf.gz	http://dec2016.archive.ensembl.org/biomart/martview/ (Ensembl Genes 87, Horse genes (EquCab 2))	*NA*	http://hgdownload.soe.ucsc.edu/goldenPath/equCab2/database/chr1_rmsk.txt.gz (…) http://hgdownload.soe.ucsc.edu/goldenPath/equCab2/database/chrX_rmsk.txt.gz
**Pig**	Sscrofa10.2	ftp://ftp.ensembl.org/pub/release-84/gtf/sus_scrofa/Sus_scrofa.Sscrofa10.2.84.gtf.gz	http://mar2016.archive.ensembl.org/biomart/martview/ (Ensembl Genes 84, Sus scrofa genes (Sscrofa10.2))	http://hgdownload.cse.ucsc.edu/goldenPath/susScr3/database/cpgIslandExt.txt.gz	http://hgdownload.cse.ucsc.edu/goldenPath/susScr3/database/rmsk.txt.gz
**Mouse**	GRCm38	ftp://ftp.ensembl.org/pub/release-83/gtf/mus_musculus/Mus_musculus.GRCm38.83.gtf.gz	http://dec2015.archive.ensembl.org/biomart/martview/ (Ensembl Genes 83, Mouse genes (GRCm38.p4))	http://hgdownload.soe.ucsc.edu/goldenPath/mm10/database/cpgIslandExt.txt.gz	http://hgdownload.soe.ucsc.edu/goldenPath/mm10/database/rmsk.txt.gz
**Human**	GRCh38	ftp://ftp.ensembl.org/pub/release-87/gtf/homo_sapiens/Homo_sapiens.GRCh38.87.gtf.gz	http://dec2016.archive.ensembl.org/biomart/martview/ (Ensembl Genes 87, Human genes (GRCh38.p7))	http://hgdownload.soe.ucsc.edu/goldenPath/hg38/database/cpgIslandExt.txt.gz	http://hgdownload.soe.ucsc.edu/goldenPath/hg38/database/rmsk.txt.gz

NA: not available

Table S2. *In silico* characterization of bovine reduced restriction (RR) genomes generated using different size selection criteria

Size selection (bp)	No	0-250	40-290	80-330	120-370	160-410	
RR genome size (Mb)	2,670	71	79	83	85	86	Regions targeted by the microarray
Per cent of whole genome	100.0	2.7	3.0	3.1	3.2	3.2	
Number of MspI fragments	1,990,837	810,994	585,584	456,504	376,199	317,974	
Number of CpG sites	27,540,276	3,588,657	3,454,028	3,127,005	2,854,907	2,666,727	
Percent of total genomic CpG sites	100.0	13.0	12.5	11.4	10.4	9.7	
Percent in 3'UTR	0.4	0.6	0.6	0.6	0.6	0.6	0.1
Percent in 5'UTR	0.2	0.5	0.4	0.3	0.2	0.2	0.1
Percent in exon	4.6	9.2	8.7	7.6	6.4	5.4	0.4
Percent in intron	31.1	30.7	32.0	33.0	33.8	34.5	5.4
Percent in intergenic	57.0	46.9	48.3	50.1	51.7	52.7	16.9
Percent in promoter-TSS	4.8	9.7	7.7	6.1	5.0	4.3	76.6
Percent in TTS	1.9	2.4	2.4	2.4	2.3	2.3	0.6
Percent in CpG islands	13.4	31.4	22.9	16.1	11.5	8.3	51.0
Percent in overlapping repeats	61.9	26.2	31.2	37.8	43.4	47.9	48.0

For comparison with RR genomes, the regions targeted by the microarray used for MeDIP-chip were annotated using the same pipeline. The genome features are shaded in gray for these regions.

Table S3. Comparison of RR genomes obtained with a 40-290 bp selection size window in different species

Species	Human	Mouse	Pig	Sheep	Horse	Cattle
Whole genome size (Gb)	3.1	2.7	2.8	2.6	2.5	2.7
Total number of CpG sites	29,345,332	21,867,837	30,460,432	26,376,870	29,873,125	27,203,575
CpG sites per Mb whole genome	9,466	8,099	10,840	10,068	12,094	10,075
RR genome size (Mb)	99	56	106	75	109	79
Number of MspI fragments in RR genome	738,215	400,739	782,851	538,822	844,221	585,584
Percent of whole genome	3.2	2.0	3.8	2.9	4.4	3.0
Number of CpG sites in RR genome	4,140,424	1,914,962	5,132,906	3,310,947	4,854,816	3,454,028
Percent of total CpG sites	14.1	8.8	16.9	12.6	16.3	12.5
CpG sites per Mb RR genome	41,992	34,442	48,424	43,912	44,540	43,611
CpG enrichment ratio	4.4	4.3	4.5	4.4	3.7	4.2

The CpG enrichment ratio was obtained by dividing the number of CpG sites per Mb RR genome by the number of CpG sites per Mb whole genome.

Table S4. Primers and PCR conditions used to generate the pyrosequencing templates

Primer name	Gene symbol (EMBL accession number)	Primer sequence (5'-3')	Size of the product	Hybridization temperature	$MgCl_2$ concentration
**bLSM4_bis_F1**	*LSM4* (ENSBTAG00000008578)	GTTTTGGTGGTTAGTTTTTTG	266 bp	60°C	2 mM
**bLSM4_bio_R1**		AATTAAAATCCTAACTTTATCCCTC			
**bDDX4_bis_F1**	*DDX4* (ENSBTAG00000008871)	GTTGGGATGATTTTTGTATTGGGAAAAG	324 bp	58°C	1.5 mM
**bDDX4_bio_R1**		CCACCATCAACCTTATACCCCCAAAC			
**bSYCP3_bis_F1**	*SYCP3* (ENSBTAG00000002492)	GGTTAAGAGTAGTTTTTGGTTTAGAT	318°C	56°C	1.5 mM
**bSYCP3_bio_R1**		ATCAACAACCTCACAAAATTCTTC			
**bBTSAT4_bis_F1**	*NA* (bovine satellite)	TGTAGATTGGGGATAGGAGAGTTAG	380 bp	60°C	3 mM
**bBTSAT4_bio_R1**		CCCTCCTAATCTAAACAAAAAAATC			

The primer bDDX4_bio_R1 contains a CpG site. To avoid the biased amplification of the methylated/unmethylated allele, the underlined base, originally a G, has been mutated. NA: not available.

Table S5. Pyrosequencing primers

Name	Sequence (5'-3')	Gene symbol (EMBL accession number)	Template	CpGs
**bLSM4_pyr1**	GAGTAGTTTGTTTGG	*LSM4* (ENSBTAG00000008578)	bLSM4_bis_F1 x bLSM4_bio_R1	#1-9
**bLSM4_pyr3**	TTGTTGTTTAAAGAG			#10-15
**bLSM4_pyr5**	GGAGGTGAATTAAGG			#16
**bDDX4_pyr2**	TTTATTTTTAGTTTTTTTTATTTTA	*DDX4* (ENSBTAG00000008871)	bDDX4_bis_F1 x bDDX4_bio_R1	#1-8
**bSYCP3_pyr1**	GAGGATAGTAGTTAATGTTTT	*SYCP3* (ENSBTAG00000002492)	bSYCP3_bis_F1 x bSYCP3_bio_R1	#1-2
**bSYCP3_pyr2**	TTGAAGTGTTTATTT			#3-6
**bSYCP3_pyr4**	GGGAGAAAAGTTAGTTT			#9-12
**bBTSAT4_pyr3**	ATTTATAGGTTGGAG	Bovine satellite	bBTSAT4_bis_F1 x bBTSAT4_bio_R1	#1
**bBTSAT4_pyr4**	TTTTATTAAGAGGGG			#4-6
**bBTSAT4_pyr5**	GTTTGGAATGTTTT			#9-12

The last column refers to the CpG positions indicated Fig. 6b. The primers bDDX4_pyr2 and bSYCP3_pyr1 contain a CpG site. To avoid the biased elongation of the methylated/unmethylated allele, the underlined base, originally a C, has been mutated.

Table S6. Library characterization, mapping efficiency on the bovine genome (UMD3.1), coverage and average methylation in RRBS libraries

Sample	spz32	spz34	mono1	mono2	F029	F5538
**Number of read pairs (million)**	26.8	29.9	27.7	22.6	40.8	33.7
**Uniquely mapped reads (%)**	35.5	36.9	38.5	36.8	35.8	36.4
**Ambiguous reads (%)**	55.6	54.3	53.1	52.5	50.0	48.6
**Unmapped reads (%)**	8.9	8.8	8.4	10.7	14.2	15.0
**Bisulfite conversion rate (%)**	99.7	99.8	99.8	99.9	99.5	99.6
**Methylation, all CpGs (%)**	54.0	53.8	60.6	59.6	49.7	49.0
**Methylation, CpGs 5-500 (%)**	51.4	52.1	58.3	56.9	48.3	47.6
**Methylation, CpGs >500 (%)**	23.9	21.2	81.6	81.2	67.0	66.3
**Number of CpGs 5-500**	2,310,551	2,438,658	2,537,411	2,318,185	2,673,984	2,609,798
**Number of CpGs >500**	8,254	10,258	8,168	6,807	11,364	8,949

Bisulfite conversion rate was estimated from the unmethylated cytosine added *in vitro* during the end-repair step. CpGs 5-500 comprise all CpGs covered by 5 to 500 reads for each sample, and CpGs >500 comprise all CpGs covered by more than 500 reads for each sample.

Table S7. Results of the comparisons between tissues by RRBS

Comparison	Sperm *vs* Fibroblasts	Sperm *vs* Monocytes	Fibroblasts *vs* Monocytes
**Number of methylKit DMCs**	450,959	298,874	239,017
**Number of obvious DMCs**	100,686	128,477	32,883
**Number of obvious DMCs added**	12	27	19

The number of obvious DMC added corresponds to the obvious DMCs that were not identified using methylKit.

Table S8. Mapping efficiency on a Repbase artificial bovine genome, coverage and average methylation in RRBS libraries

Sample	spz32	spz34	mono1	mono2	F029	F5538
Uniquely mapped reads (%)	25.8	25.3	25.0	23.5	22.8	21.2
Unmapped reads (%)	74.2	74.7	75.0	76.5	77.2	78.8
Methylation, all CpGs (%)	39.1	38.0	56.2	58.4	44.7	46.0
Methylation, CpGs 5-500 (%)	47.2	45.5	64.0	65.8	49.1	51.2
Methylation, CpGs >500 (%)	25.7	25.3	56.7	60.2	39.1	44.2
Number of CpGs 5-500	557	541	569	513	559	536
Number of CpGs >500	370	383	391	344	389	382
Hypomethylated CpGs 5-500 fraction	0.29	0.30	0.16	0.12	0.25	0.19
Intermediate CpG 5-500 fraction	0.49	0.49	0.40	0.44	0.54	0.61
Hypermethylated CpGs 5-500 fraction	0.23	0.21	0.44	0.44	0.21	0.21
Hypomethylated CpGs >500 fraction	0.65	0.64	0.14	0.02	0.42	0.25
Intermediate CpG >500 fraction	0.28	0.31	0.50	0.65	0.44	0.61
Hypermethylated CpGs >500 fraction	0.07	0.05	0.36	0.33	0.13	0.14

CpGs 5-500 comprise all CpGs covered by 5 to 500 reads for each sample, and CpGs >500 comprise all CpGs covered by more than 500 reads for each sample. Hypomethylated, intermediate and hypermethylated CpGs 5-500 (resp. CpGs >500) fractions: fractions of CpGs 5-500 (resp. CpGs >500) with average methylation <20%, [20%; 80%], and >80%, respectively.

Supplementary Figures

Figure S1

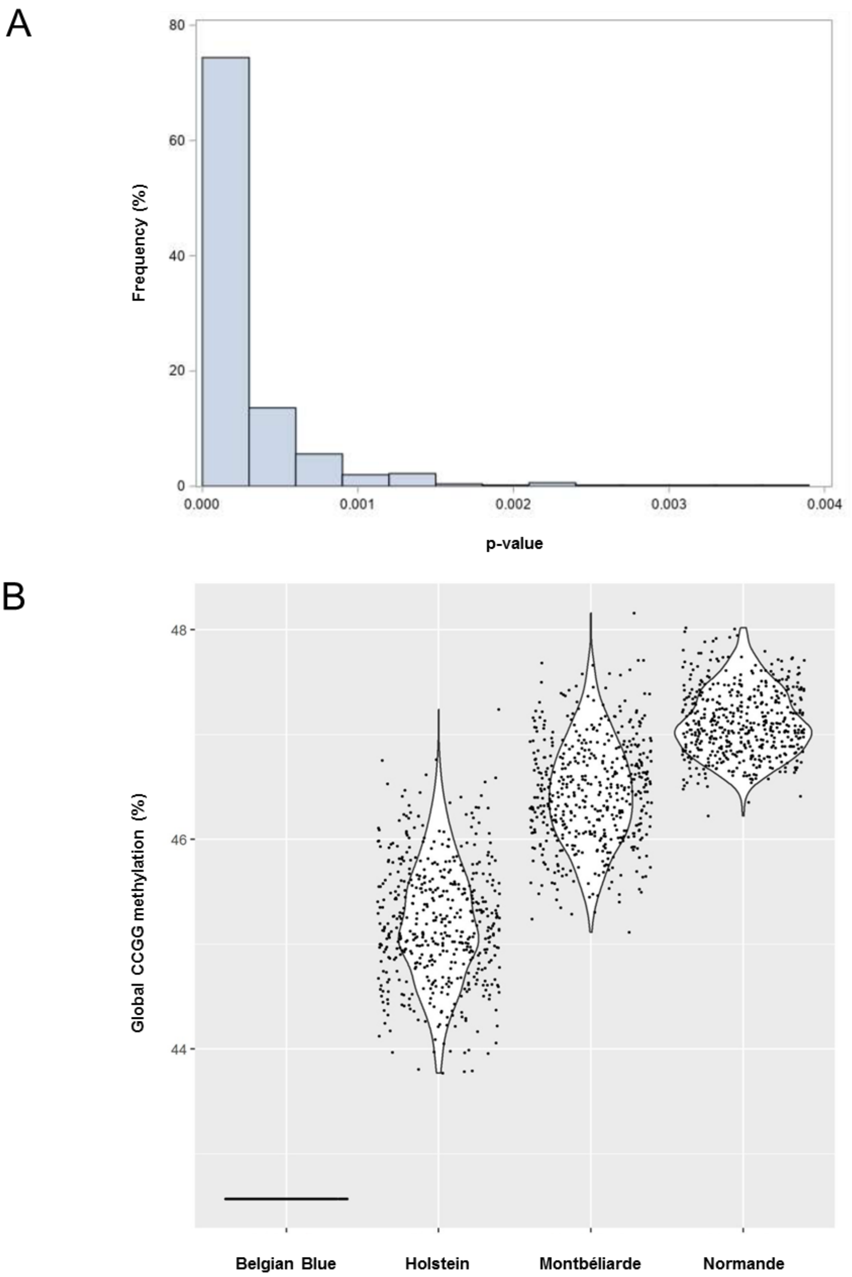

Bootstrap analysis of global CCGG methylation in bull sperm from four different breeds. To check whether the breed-related differences observed Fig. 1b were not due to an unbalanced number of bulls from each breed, 14 bulls from each breed were randomly selected and an analysis of variance was conducted on this subsample. All the procedure was performed using SAS version 9.4. (A) Distribution of the p-values for 500 subsamples, demonstrating a significant effect of the breed whatever the subsample. (B) For each subsample, the mean was calculated in each breed from the 14 methylation percentages. The figure shows the distribution of 500 means in each breed, which is very similar to that obtained with the whole sample (Fig. 1b). Each dot represents one mean, and the violin plots indicate the density of the distribution in each breed.

Figure S2

Bull 1: comparison of Log R ratio between sperm and blood

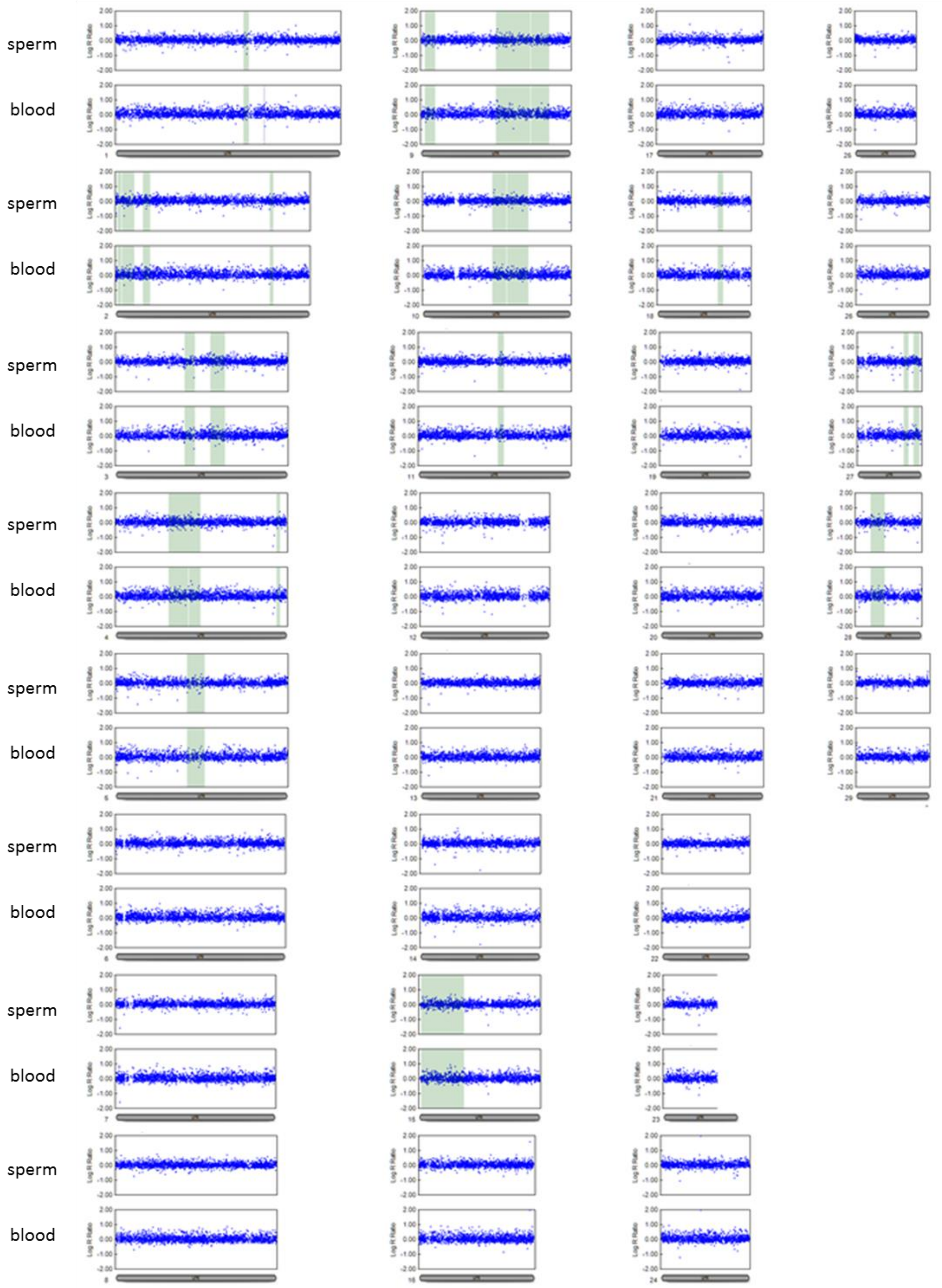

Figure S2 (continued)

Bull 2: comparison of Log R ratio between sperm and blood

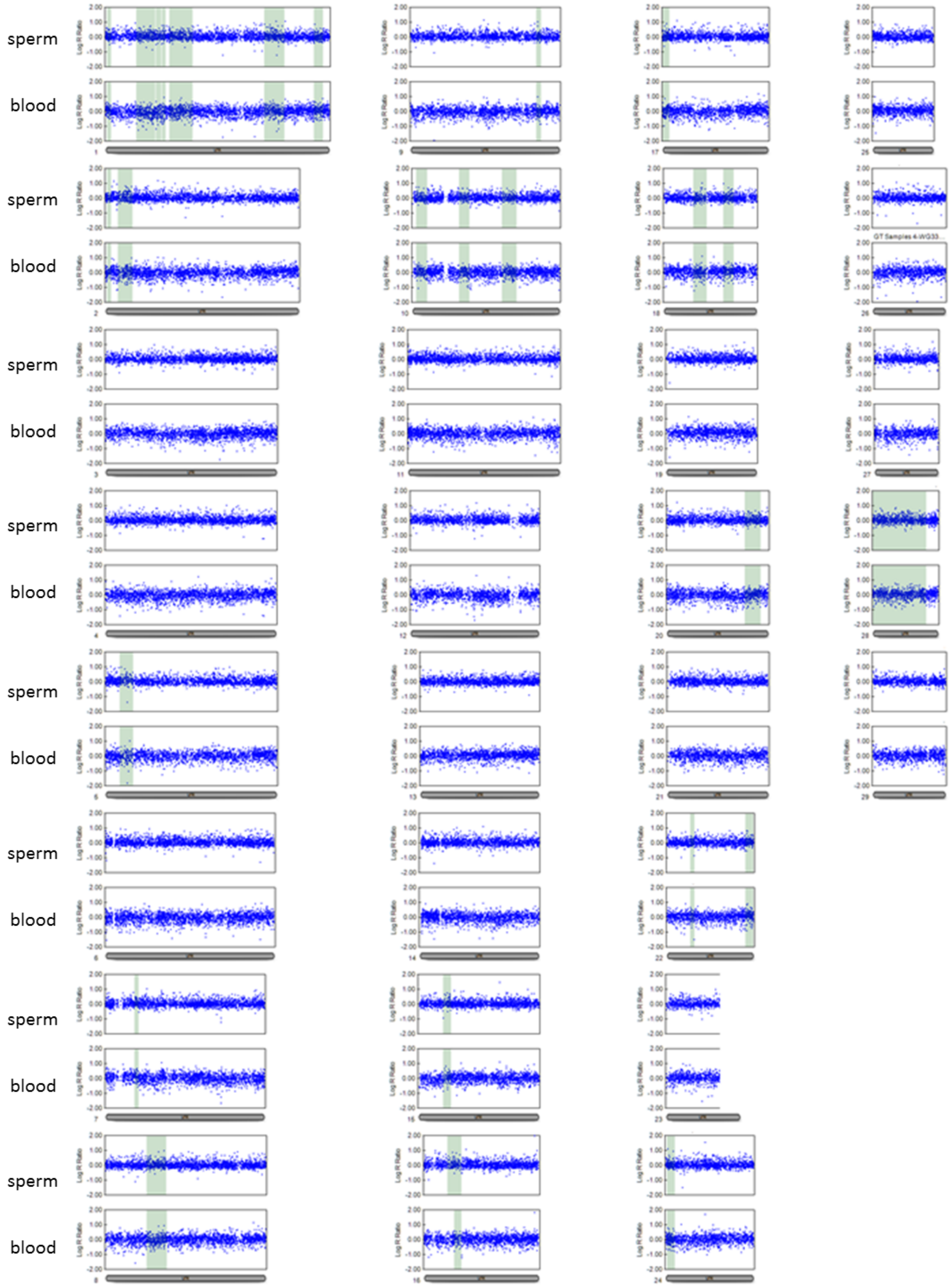

Figure S2 (continued)

	Sperm			Blood			
	Call Rate	Calls	No Calls	Call Rate	Calls	No Calls	Genotype discrepancies
Bull 1	0.9979373	55154	114	0.9978468	55149	119	29
Bull 2	0.9980459	55160	108	0.9981725	55167	101	21

Genotyping of bull sperm and blood samples. For two bulls, CNV profiles were compared between tissues since any difference might be indicative of preferential extraction. The plots on the two precedent pages show the log R ratio (LRR) along each chromosome. The regions identified by the cnvPartition algorithm as having aberrant copy numbers are delineated by a light green background. A LRR segment above zero typically indicates a copy number gain, whereas a LRR segment mean below zero suggests a copy number loss. For each bull, LRR ratio plots were similar and no gross discrepancies could be observed between sperm and blood samples, while different bulls exhibited contrasting profiles. The table indicates call rates (% of called SNPs) and genotypes for the two bulls. Similar call rates were observed in sperm and blood samples and the same genotype was obtained from blood and sperm DNA for each bull. A few (0.05%) genotype discrepancies were observed between the two cell types, mostly due to a missing genotype in one sample. This suggests that DNA extraction from sperm does not induce any bias compared to blood DNA extraction.

Figure S3

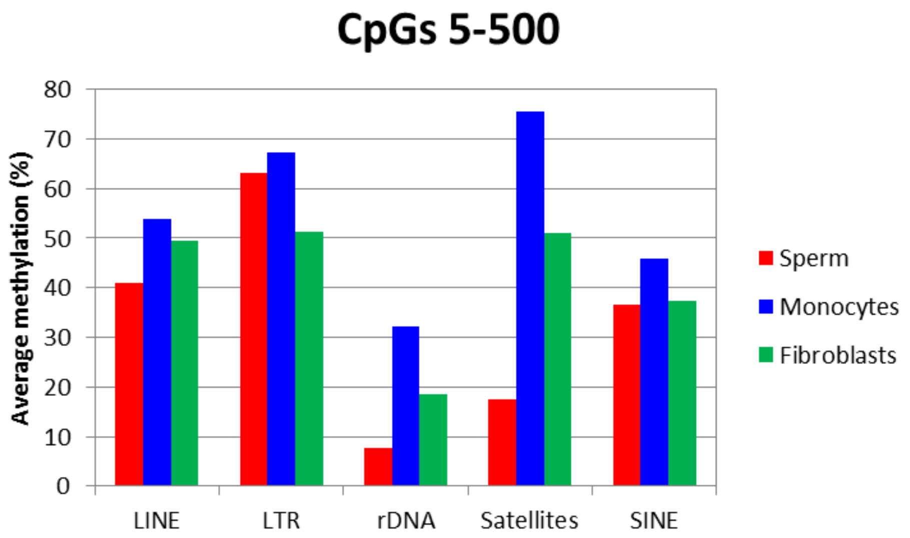

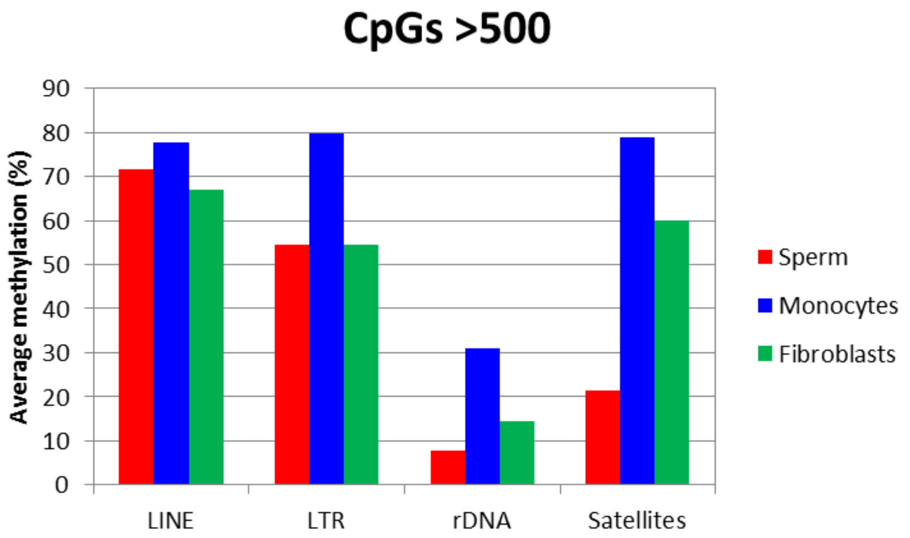

Average methylation percentage for CpGs 5-500 and CpGs >500 in each cell type, in reads uniquely aligned on a Repbase bovine artificial genome. For each RRBS sample, reads were mapped on an artificial genome containing one copy of each bovine repeat as defined in the Repbase database. The average percentage of methylation was then calculated individually for each consensus repeat, from either CpGs 5-500 or CpGs >500. The figures shows this average methylation percentages in each family of repeat.

Figure S4

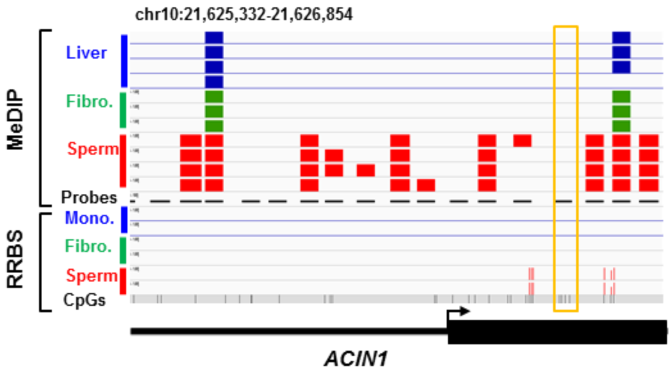

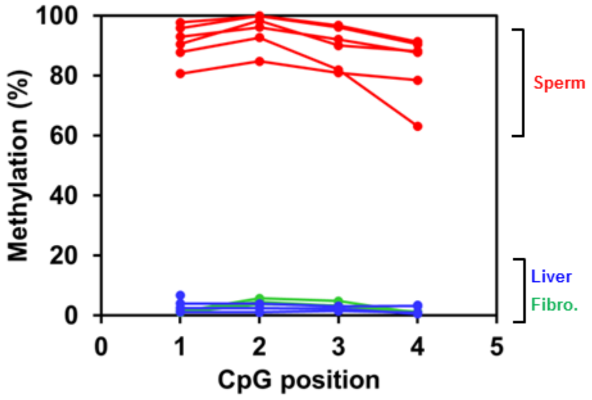

Primer name	Gene symbol (EMBL accession number)	Primer sequence (5'-3')	Size of the product	Hybridization temperature	$MgCl_2$ concentration
**bACIN1_bis_F1**	*ACIN1* (ENSBTAG00000011571)	GTTGGGTAAGTGGTAAGATGGTTT	258 bp	60°C	3 mM
**bACIN1_bio_R1**		AAATACAACAACTAAAAATCCAAAAA			

Pyrosequencing of CpGs hypermethylated in sperm. Upper panel: IGV browser view of the gene region targeted for pyrosequencing (*ACIN1* gene). The orange box delineates the four CpGs analyzed by pyrosequencing. Lower panel: methylation percentages of the CpGs assayed by pyrosequencing in sperm (n=6), fibroblasts (n=3) and liver (n=4). The difference between sperm and somatic cells is significant at every position ($p<0.05$, permutation test). The table indicates the primers and PCR conditions used to generate the pyrosequencing template. The following primer was used for pyrosequencing: 5'-GAGAGATTAATTTAG-3'.
